# The impact of health information technology on prescribing errors in hospitals: a systematic review and behaviour change technique analysis

**DOI:** 10.1186/s13643-020-01510-7

**Published:** 2020-12-03

**Authors:** Joan Devin, Brian J. Cleary, Shane Cullinan

**Affiliations:** 1 RCSI School of Pharmacy and Biomolecular Sciences, Royal College of Surgeons in Ireland, Dublin 2, Ireland; 2 Department of Pharmacy, The Rotunda Hospital, Parnell Square, Dublin 1, Ireland

**Keywords:** HIT, CPOE, ePrescribing, Prescribing errors, Technology-generated errors, Behaviour change techniques, BCTTv1, Synthesis without meta-analysis

## Abstract

**Background:**

Health information technology (HIT) is known to reduce prescribing errors but may also cause new types of technology-generated errors (TGE) related to data entry, duplicate prescribing, and prescriber alert fatigue. It is unclear which component behaviour change techniques (BCTs) contribute to the effectiveness of prescribing HIT implementations and optimisation. This study aimed to (i) quantitatively assess the HIT that reduces prescribing errors in hospitals and (ii) identify the BCTs associated with effective interventions.

**Methods:**

Articles were identified using CINAHL, EMBASE, MEDLINE, and Web of Science to May 2020. Eligible studies compared prescribing HIT with paper-order entry and examined prescribing error rates. Studies were excluded if prescribing error rates could not be extracted, if HIT use was non-compulsory or designed for one class of medication. The Newcastle-Ottawa scale was used to assess study quality. The review was reported in accordance with the PRISMA and SWiM guidelines. Odds ratios (OR) with 95% confidence intervals (CI) were calculated across the studies. Descriptive statistics were used to summarise effect estimates. Two researchers examined studies for BCTs using a validated taxonomy. Effectiveness ratios (ER) were used to determine the potential impact of individual BCTs.

**Results:**

Thirty-five studies of variable risk of bias and limited intervention reporting were included. TGE were identified in 31 studies. Compared with paper-order entry, prescribing HIT of varying sophistication was associated with decreased rates of prescribing errors (median OR 0.24, IQR 0.03–0.57). Ten BCTs were present in at least two successful interventions and may be effective components of prescribing HIT implementation and optimisation including prescriber involvement in system design, clinical colleagues as trainers, modification of HIT in response to feedback, direct observation of prescriber workflow, monitoring of electronic orders to detect errors, and system alerts that prompt the prescriber.

**Conclusions:**

Prescribing HIT is associated with a reduction in prescribing errors in a variety of hospital settings. Poor reporting of intervention delivery and content limited the BCT analysis. More detailed reporting may have identified additional effective intervention components. Effective BCTs may be considered in the design and development of prescribing HIT and in the reporting and evaluation of future studies in this area.

**Supplementary Information:**

The online version contains supplementary material available at 10.1186/s13643-020-01510-7.

## Background

Medication errors cost the global economy an estimated $42 billion each year [1] and occur most frequently during the prescribing stage of the medication use process [2]. Health information technology (HIT) is well-documented as a means to improve patient safety by reducing prescribing errors and associated adverse drug events [3, 4]. However, Cresswell et al. [5] describe a ‘long road’ of ongoing user engagement and evaluation, from the initial HIT implementation to eventual optimisation of a system.

Black et al. [6] define three categories of prescribing HIT: computerised provider order entry (CPOE), electronic prescribing (ePrescribing), and clinical decision support (CDS). CPOE allows prescribers to enter, modify, and transmit medication and other orders electronically, typically within a central electronic health record (EHR). ePrescribing involves the electronic ordering and transmission of prescriptions via a standalone or EHR-integrated system. CDS may exist as standalone knowledge support without ordering functions or be integrated with CPOE or ePrescribing systems. In Ireland, HIT is in the early stages of adoption [7]. The most established system is the Maternal and Newborn Clinical Management System (MN-CMS), an EHR with CPOE and integrated CDS. MN-CMS is currently used in four Irish maternity units and 40% of annual births, with a national phased rollout planned [7].

Previous systematic reviews of prescribing HIT have demonstrated a reduction in medication errors in comparison to paper-based ordering [8–10]. However, technology-generated errors (TGE) have also been linked with these complex sociotechnical interventions [11]. Common TGE include data entry errors, duplicate orders, and override of critical alerts due to user alert fatigue [11–14]. A review of prescribing errors caused by CPOE recommended that human factor principles, or behavioural influences, be considered when designing or adapting HIT [12]. The Agency for Healthcare Research and Quality similarly suggests that studies of HIT should fully report the intervention context and propose the development of behaviour theory-based taxonomies to assist with this [15].

A behaviour change technique (BCT) is ‘an observable, replicable, and irreducible component of an intervention designed to alter or redirect causal processes that regulate behaviour’ [16]. BCTs may be identified and classified using the behaviour change technique taxonomy version 1 (BCTTv1), an extensive taxonomy of 93 BCTs, organised into 16 hierarchical clusters [16, 17]. The BCTTv1 was developed through international, interdisciplinary consensus methods in response to a need for a usable, replicable, and standardised BCT taxonomy [16].

Retrospective application of the BCTTv1 in a systematic review allows for comparison and synthesis of evidence across studies in a structured manner. The benefits of this analysis include the ability to identify the explicit mechanisms of behavioural change reported in successful interventions, and by doing so avoid any implicit assumptions of what works [18]. While this is still an emerging approach, previous systematic reviews have successfully identified effective BCTs for smoking cessation [19], interventions in diabetes care [20], prevention and management of childhood obesity [21], and deprescribing interventions [22, 23], thereby maximising the potential success of future implementation studies in these areas. While BCTs to facilitate nurses’ use of HIT for medication administration have been previously identified [24], to our knowledge, a defined and usable set of specific BCTs for prescribers has not yet been constructed.

A systematic review and BCTTv1 analysis of the impact of HIT on prescribing errors may inform a dynamic implementation framework for prescribing HIT in Ireland. As eHealth and HIT in Ireland expand, empirical findings may in turn be applied on a larger scale to benefit systems worldwide.

The aims of this systematic review are to (i) identify and quantitatively summarise the HIT that reduces prescribing errors in hospitals and (ii) subsequently identify the BCTs associated with effective interventions.

## Methods

The PRISMA [25] and Synthesis Without Meta-Analysis (SWiM) [26] reporting guidelines were used to structure this review (see Additional file 1).

### Inclusion criteria

Studies were included if they (i) reported on the impact of HIT on prescribing errors in hospitals and (ii) used an experimental or observational design. No restrictions were applied to population or timeframe. Studies in English language with full availability were considered.

Studies were excluded if they did not report rates of prescribing errors and reported on prescription completeness errors only or if prescribing error rates could not be extracted. Studies evaluating non-compulsory use of HIT were excluded, as their results were unlikely to be reflective of site error rates. While we included studies that used error rates of particular medications if the outcomes were decided by the study authors a priori, studies that focused on HIT designed for prescribing a single class of medication (e.g. anti-neoplastic or anti-retroviral) were excluded to avoid variability related to clinical or contextual factors.

### Search strategy

The Cochrane Library and PROSPERO international prospective register of systematic reviews were first searched for similar reviews or registered protocols to avoid replication. A protocol was not registered for this review. The databases CINAHL, EMBASE, MEDLINE, and Web of Science were then searched using a combination of keywords, with no publication date restrictions to November 2018. Searches were updated in May 2020. Keywords were selected and revised appropriately with the assistance of a medical librarian (see Additional file 2). Additional citations were sourced from the bibliographies of review articles and key journals.

### Data extraction and critical appraisal

Titles and abstracts were screened by JD and SC against the inclusion criteria. Disagreements surrounding studies for inclusion were resolved by discussion. After removal of duplicates, full papers were reviewed by JD. A data extraction form was used to collate information on study characteristics, population, intervention, setting, software manufacturer, error detection methods, and prescribing error rates. We did not examine harm as a result of prescribing errors. Where no absolute numbers were provided for error rates, this was calculated based on given data. For time series analysis designs, the last reported measurement was used as post-intervention data. The Newcastle-Ottawa scale (NOS) for assessing the quality of non-randomised studies was used to assess the risk of bias [27]. Studies were judged for methodological quality but not excluded from data analysis if they otherwise met inclusion criteria to best capture BCTs and answer the review question. Data extraction and critical appraisal was performed by JD.

### Statistical analysis

A meta-analysis was not planned due to the anticipated diversity of included studies necessary to capture BCTs in varying contexts. Units of exposure varied across the studies, but the number of medication orders was used where possible, as in previous reviews on this topic [8, 9, 28]. Review Manager version 5.3 [29] was used to calculate OR with 95% confidence intervals for the included studies by comparing prescribing error data pre- and post-intervention. This was used as a standardised metric by which to assess intervention effectiveness in the studies. Following the SWiM guideline [26] and McKenzie and Brennan’s recommendations for data presentation of alternative quantitative synthesis [30], forest plots were used to present the impact of CPOE and ePrescribing on prescribing errors versus paper-based ordering to allow for graphical comparison of individual effects and to visually assess the likelihood of statistical heterogeneity. The impact of CDS on prescribing errors was presented in the forest plots as a subgroup analysis. Descriptive statistics were used to combine OR. The median OR with interquartile (IQR) ranges is reported for (i) all studies, (ii) by risk of bias, and (iii) by HIT type and presence of CDS. Box-and-whisker plots were used to examine informally whether the distribution of effects differed by the overall risk of bias assessment. Spearman’s correlation determined whether the frequency of coded BCTs affected the OR of prescribing errors in the interventions. All statistical analyses were performed in Stata version 15.1 [31].

### Behaviour change technique coding

BCT coding was performed independently by two reviewers; JD coded all studies, and SC coded a subset of the studies. JD completed online training on the BCTTv1, while SC had previously used the BCTTv1 and is an experienced qualitative researcher. The target behaviour was optimisation of prescribing in order to reduce prescribing errors. The description of the interventions in each study was read line-by-line and analysed for the clear presence of BCTs, using guidelines by Michie et al. [32]. Preliminary coding was performed by JD, and then a coding manual constructed for use by both researchers. This included a subset of BCTs and their definitions, with HIT-specific examples (see Additional file 3). For each study, identified BCTs were documented and categorised alongside supporting evidence from the text. Identified BCTs were coded once only per study, regardless of the amount of times that they were identified within the text. BCTs were also sub-categorised into ‘HIT implementation’ and ‘HIT optimisation’, based on the context provided. Disagreements surrounding the inclusion of a BCT were resolved by discussion. If there was uncertainty as to whether a BCT was present in a study, it was coded as absent. NVivo version 12 was used for all coding [33].

### Assessment of BCT effectiveness

A BCT was considered to be a facilitator of the target behaviour if it was present in two or more successful studies. These criteria have previously been used in studies determining BCT effectiveness [19, 21, 34]. Following the method described by Martin et al. [21], the BCT percentage effectiveness ratio (ER) was calculated for each potentially effective BCT. To calculate the ratio, the number of times each BCT was coded in an effective intervention divided by the number of times it was coded in all studies. The ER provides a weighted measure for comparison of BCTs and has been used in studies retrospectively identifying the impact of BCTs [35, 36].

## Results

### Search results

**Fig. 1:**
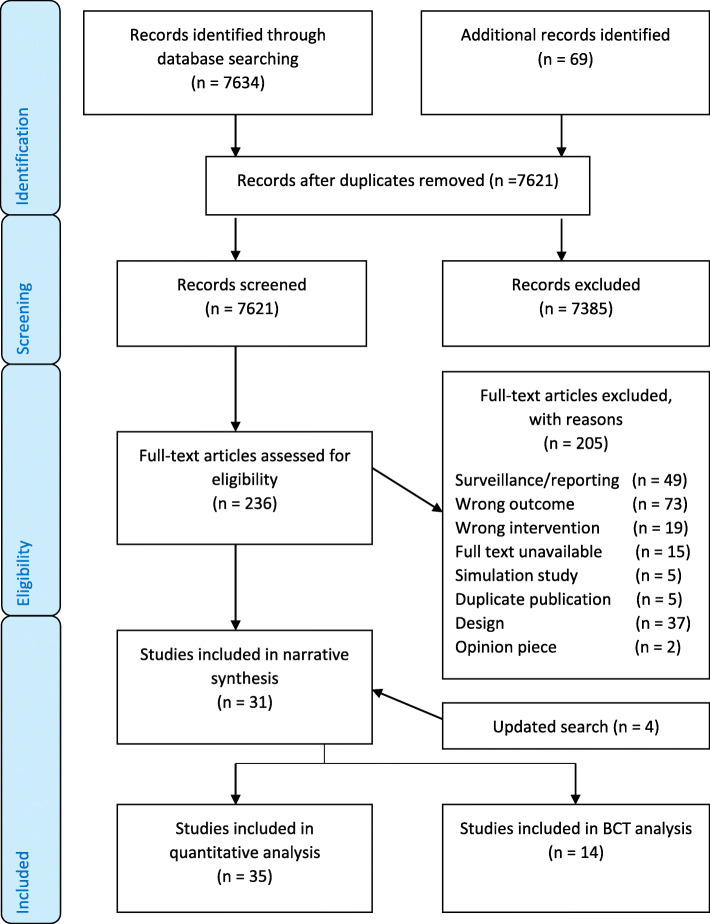
PRISMA summary of evidence search and selection

The searches identified 7621 potentially relevant citations after duplicates were removed. After full-text screening, 31 studies met the inclusion criteria. Four additional studies were included after searches were updated in May 2020. A PRISMA flow chart was used to document the study selection process (see Fig. 1). Fourteen studies were included in the BCT analysis.

### Study characteristics

Twenty studies focused on adults [37–56], ten on a paediatric or neonatal population [57–66], and five included a mixed population [67–71] (see Table 1). Ten studies were based in the US [37, 41, 42, 48, 54, 57, 58, 64, 67, 70], eight in the UK [39, 45, 49, 50, 53, 60, 65, 66], seven in Europe [40, 43, 44, 46, 52, 55, 59], four in the Middle East [47, 61, 62, 69], three in Australia [38, 56, 68], two in Asia [51, 71], and one in Canada [63]. Twenty-two studies used a pre-post design [38, 42, 44–47, 49, 50, 53–55, 57, 58, 60, 63–65, 67–71], nine used a time series analysis design [39–41, 48, 52, 59, 61, 62, 66], three used similar groups as controls [37, 43, 51], and Westbrook et al. [56] used a difference in differences design.

**Table 1:** Characteristics and summary of findings of the included studies

Study (country)	Population	Prescribing-associated HIT	Study design	Hospital setting	Sample	Error detection method	Baseline error rate (%)	Technology-generated errors (TGE) detected
Abbass 2011 [37] (USA)	Adult	CPOE (commercial)	Control group	All areas	**1110** (orders)	Chart review	20.8	Lack of CDS led to allergy/DDI errors.
Ali 2010 [39] (UK)	Adult	CPOE (commercial)	Time series analysis	ICU	**14721** (prescriptions)	Routine pharmacist review; Chart review	16.7	Allergy alert did not fire if the allergy field was not already completed by the prescriber.
Al-Sarawi 2019 [38] (Australia)	Adult	ePrescribing (EP) (commercial)	Pre-post	All areas	**4689** (orders)	Chart review	67.7	Duplicate orders increased post-CPOE.
Armada 2014 [40] (Spain)	Adult	CPOE (commercial)	Time series analysis	ICU	**5729** (orders)	Routine pharmacist review	44.8	Selection errors made while searching for drugs on drop-down menus.
Bates 1998 [42] (USA)	Adult	CPOE (homegrown)	Pre-post	2 medical wards; 2 surgical wards; 2 ICUs	**24453** (pt. days)	Routine pharmacist review; chart review	5.0	Increase in therapeutic duplication of sedating drugs, which the CPOE did not prevent.
Bates 1999 [41] (USA)	Adult	CPOE (homegrown)	Time series analysis	3 medical units	**7985** (pt. days)	Routine pharmacist review; chart review; medication order review	1.7	Missed dose errors (not main outcome of interest) increased with CPOE.
Bizovi 2002 [67] (USA)	Adult/paediatric	EP (commercial)	Pre-post	ED	**3920** (prescriptions)	Routine pharmacist review; medication order review	2.3	Free-text electronic prescriptions had a higher rate of error than the pick-list prescriptions.
Boling 2005 [57] (USA)	Paediatric	CPOE (commercial)	Pre-post	All areas	**21253** (orders)	Trigger tool methodology; chart review; voluntary error reports	0.1 (opioids)	None found.
Colpaert 2006 [43] (Belgium)	Adult	CPOE (commercial)	Prospective controlled trial	3 units in an ICU	**2510** (prescriptions)	Chart review; medication order review	27.0	CPOE errors were mostly duplicate prescriptions.
Cordero 2004 [58] (USA)	Neonatal	CPOE (commercial)	Pre-post	NICU	**211** (patients)	Chart review	12.6 (gentamicin)	None found.
Delgado Silveira 2007 [44] (Spain)	Adult	EP (commercial)	Pre-post	2 medical units	**4814** (prescriptions)	Routine pharmacist review	94.2	Drug interaction errors increased with CPOE, this was not significant.
Donyai 2008 [45] (UK)	Adult	EP (commercial)	Pre-post	Surgical ward	**4803** (orders)	Routine pharmacist review; chart review; medication order review	3.8	Selection errors were found post-EP. 1 wrong-patient error post-EP, authors uncertain if TGE.
Hernandez 2015 [46] (France)	Adult	CPOE (commercial)	Pre-post	Orthopaedic unit	**2981** (orders)	Chart review; Direct observation	30.1	Duplicate orders increased with CPOE.
Hitti 2017 [47] (Lebanon)	Adult	EP (homegrown)	Pre-post	ED	**2883** (prescriptions)	Chart review	67.7	Duplicate errors increased with CPOE.
Hodgkinson 2017 [68] (Australia)	Adult/paediatric	EP (commercial)	Pre-post	ED and OPD	**1289** (orders)	Routine pharmacist review; medication order review	95.0	50 systems-related errors post-CPOE, such as selection errors or not filling in necessary fields.
Howlett 2020 [59] (Ireland)	Paediatric	EP (commercial)	Time series analysis	PCCU	**3356** (orders)	Routine pharmacist review	10.2	Incorrect formulation and dose errors increased with EP.
Jani 2008 [60] (UK)	Paediatric	EP (commercial)	Pre-post	Nephrology OPD	**2222** (orders)	Routine pharmacist review; Chart review	7.1	Duplicate orders increased with CPOE. Wrong route, frequency, and overdose also found due to selection errors.
Kadmon 2009 [61] (Israel)	Paediatric	CPOE (commercial)	Time series analysis	PICU	**5000** (orders)	Medication order review	5.5	Prescriptions were found to be prescribed by nurses, due to doctors using computers where a nurse was already logged in.
Kazemi 2011 [62] (Iran)	Neonatal	CPOE (commercial)	Time series analysis	Neonatal unit	**4508** (medication days)	Medication order review	51.9	‘Neighbouring cell’ errors were noted, where a prescriber chose a nearby cell in error or used incorrect data to do dose calculations.
Kenawy 2019 [69] (Egypt)	Adult/paediatric	EP (commercial)	Pre-post	4 OPDs (cardiology, nephrology, paediatric, neurology)	**25057** (orders)	Voluntary error reports	28.3	Indication and omission prescribing errors increased with EP.
King 2003 [63] (Canada)	Paediatric	CPOE (commercial)	Pre-post	3 medical wards; 2 surgical wards	**12460** (patients)	Voluntary error reports	0.1	None found.
Liao 2017 [48] (USA)	Adult	CPOE (commercial)	Time series analysis	ICU	**3988** (pt. days)	Chart review	86.6	Reduction in errors only evident 2 years post-implementation.
Mahoney 2007 [70] (USA)	Adult/paediatric	CPOE (commercial)	Pre-post	All areas	**2843165** (orders)	Routine pharmacist review	0.33	Duplicate errors did not significantly decrease with CPOE.
Mills 2017 [49] (UK)	Adult	EP (commercial)	Pre-post	All areas	**318** (patients)	Chart review; medication order review	99.4	8/37 errors post- EP were selection errors on menus.
Pontefract 2018 [50] (UK)	Adult	CPOE (commercial)	Pre-post	All areas	**2422** (patients)	Trigger tool methodology; routine pharmacist review; chart review	5.0	Increase in insulin prescribing errors with CPOE in 1 site due to lack of CDS.
Potts 2004 [64] (USA)	Paediatric	CPOE (homegrown)	Pre-post	CCU	**13828** (orders)	Routine pharmacist review; medication order review	30.1	Dose errors related to trailing decimal points or missing weights occurred with CPOE.
Riaz 2014 [51] (Pakistan)	Adult	EP (homegrown)	Control group	2 OPD and 2 ED	**2040** (prescriptions)	Medication order review	52.0	Omission errors higher on EP prescriptions which caused error increase.
Rouayroux 2019 [52] (France)	Adult	CPOE (commercial)	Time series analysis	Cardiology and diabetes depts.	**3086** (pt. days)	Routine pharmacist review	12.1	Unit of use errors and duplicate orders increased with CPOE.
Shawahna 2011 [71] (Pakistan)	Adult/paediatric	EP (homegrown)	Pre-post	All areas	**32662** (orders)	Chart review; medication order review	21.7	None found.
Shulman 2005 [53] (UK)	Adult	CPOE (commercial)	Pre-post	ICU	**3465** (prescriptions)	Routine pharmacist review	6.4	Errors related to overdose increased with CPOE, with the potential to cause serious morbidity or mortality. Orders were frequently unsigned and therefore invalid.
Spencer 2005 [54] (USA)	Adult	CPOE (commercial)	Pre-post	2 medical units	**4339** (pt. discharges)	Voluntary error reports	1.4	23 reported errors caused by CPOE, including allergy errors, duplicate orders, input errors, and discrepancies when transcribing to pharmacy computer.
Van Doormaal 2009 [55] (The Netherlands)	Adult	CPOE (site 1 commercial) (site 2 homegrown)	Pre-post	4 medical wards	**1195** (patients)	Chart review; medication order review	78.6	Overriding of alerts occurred with CPOE due to alert fatigue.
Venkataraman 2016 [65] (UK)	Paediatric	EP (homegrown)	Pre-post	PCCU	**251** (prescriptions)	Routine pharmacist review	32.6	Wrong-patient error due to manual input of date of birth.
Warrick 2011 [66] (UK)	Paediatric	EP (commercial)	Time series analysis	PICU	**624** (prescriptions)	Chart review	8.8	Infusions were prescribed with no diluent or rate with EP.
Westbrook 2012 [56] (Australia)	Adult	CPOE (commercial)	Difference in differences	6 medical wards	**15194** (pt. days)	Routine pharmacist review	48.5	Selection errors occurred with CPOE.

Most studies used a combination of methods to detect prescribing errors, including routine pharmacist review of orders, retrospective chart review or medication order review, or review of voluntary error reports. A validated trigger tool methodology was used in two studies [50, 57]. While there was no consensus on definition of prescribing errors, both clinical (incorrect drug, dose, route, or frequency; drug-drug interactions; allergy or contraindication) and procedural errors (quality or completeness of prescription) were commonly evaluated together.

Twenty-seven studies evaluated commercial HIT [37–40, 43–46, 48–50, 52–54, 56–63, 66–70]. Seven studies evaluated homegrown, self-developed systems [41, 42, 47, 51, 64, 65, 71], although these varied in sophistication. One study evaluated both a commercial and a homegrown system [55]. Using the Black et al. [6] taxonomy, 21 of the studies were CPOE systems and 14 ePrescribing systems. Where there was an overlap between the characteristics of a CPOE system and an ePrescribing system, as in the HIT described by Al-Sarawi et al. [38], the described characteristics as well as the authors’ terminology were considered in order to categorise the HIT.

An EHR was integrated in 18 studies [38, 39, 41–43, 45, 48, 56–59, 61, 62, 64, 66, 67, 70, 72]. A variety of CDS was present in 18 CPOE systems, ranging from allergy and drug-drug interaction alerts to therapeutic duplication alerts, weight-based dosing, and renal function dosing [39–43, 46, 48, 50, 52, 54–58, 61, 62, 64, 70]. In contrast with CPOE systems, just three ePrescribing systems used alert-based CDS [38, 60, 68].

Delivery of the intervention was discussed in less than half of the studies [39, 40, 48, 53, 58, 59, 62, 64, 68, 70, 71], with minimal detail on the implementation itself. Of those that provided detail, eleven used formal prescriber training methods such as online or classroom learning [40, 48, 53, 58, 59, 62, 64, 68, 70, 71], while Bizovi et al. [67] used handouts only. No study explicitly stated the use of behavioural change theory in the design or implementation processes of their HIT interventions. Ali et al. [39], Cordero et al. [58], Kazemi et al. [62], Mahoney et al. [70], and Howlett et al. [59] reported end user or MDT involvement in the configuration of their commercial systems, while all of the homegrown systems were by design, site specific. See Additional file 4 for full study descriptions and characteristics.

### Risk of bias and quality assessment

The majority of studies were judged to be at medium risk of bias and evaluated subsets of a hospital population, without randomised sampling. Two studies were judged to be at low risk of bias and were published within the last 5 years. Fourteen studies were at high risk of bias due to their focus on a small population, short study period, or subjective error detection methods. As in previous systematic reviews with a BCT analysis, we did not exclude studies with high risk of bias to increase the potential capture of BCTs within study descriptions [23, 36, 73].

Due to the diversity of population and study design, there was considerable clinical heterogeneity across the studies. Forest plots were structured by type of HIT (CPOE and ePrescribing, with or without CDS); these characteristics were pre-specified during data extraction. While informal visual examination suggested that studies were observed to favour the intervention, confidence intervals for OR of the individual studies had poor overlap indicating statistical heterogeneity. Funnel plots used to visually assess publication bias across studies demonstrated asymmetry. Likely reasons for the asymmetry include the variety of populations, study sizes, error detection methods, and presence or absence of CDS as opposed to lack of reporting of negative studies. The completed risk of bias assessments are detailed in Additional file 5.

### Impact of the interventions on prescribing errors

A decrease in the rate of prescribing errors was reported in all but one study—Riaz et al. [51] compared the prescribing error rates in two hospitals (one using manual prescriptions and one using ePrescribing) and reported higher rates of prescribing errors in the hospital using ePrescribing.

**Fig. 2:**
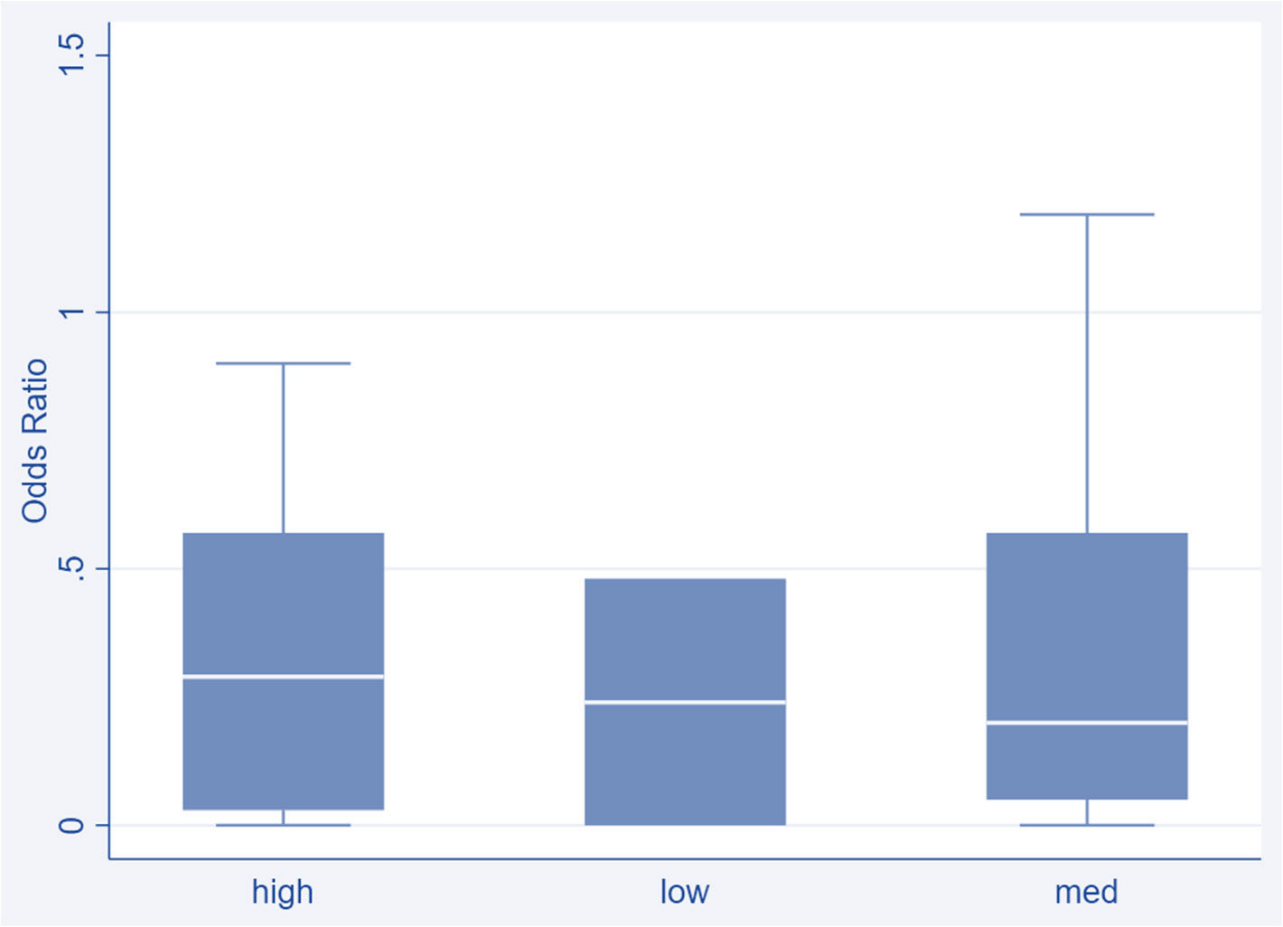
Box-and-whisker plot of the odds ratio of prescribing errors for all included studies, grouped by high risk of bias rating (*n* = 14), low risk (*n* = 2), and medium risk (*n* = 19)

The median OR for all studies comparing HIT with paper-based ordering was 0.24 (IQR 0.03–0.57, 35 studies). The median OR for studies at low risk of bias was 0.24 (IQR 0.0–0.48, 2 studies), OR for medium risk of bias was 0.20 (IQR 0.05–0.57, 19 studies), and OR for high risk of bias was 0.29 (IQR 0.03–0.57, 14 studies). This suggests that the risk of bias did not skew the overall effect estimate summary (see Fig. 2).

#### CPOE

**Fig. 3:**
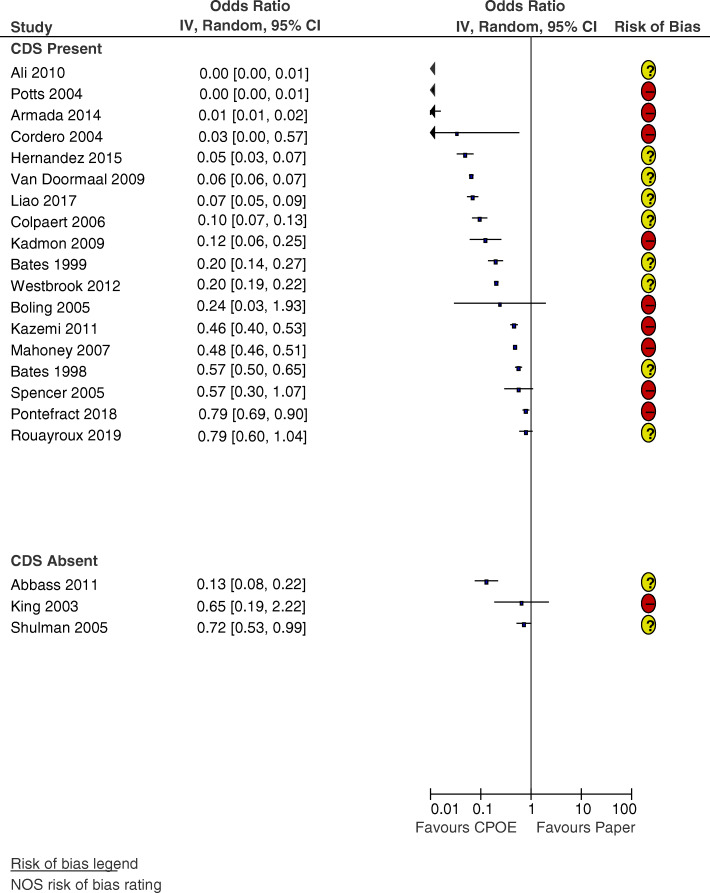
Forest plot of the odds ratio of prescribing errors for computerised provider order entry (CPOE) vs paper-based ordering, where clinical decision support (CDS) was present (*n* = 18) or absent (*n* = 3)

Seventeen studies comparing the impact of CPOE versus paper-based ordering on prescribing errors were observed to have a lower OR after the intervention (see Fig. 3). In four studies, the OR was not significant. The median OR for CPOE studies with CDS was 0.16 (IQR 0.05–0.48, 18 studies). In the three studies where CDS was absent, Abbass et al. [37] demonstrated a significant reduction in the OR of prescribing errors (0.13 [95% CI 0.08–0.22]), while the OR in King et al. [63] was non-significant (0.65 [95% CI 0.19–2.22]) and the OR of Shulman et al. [53] only marginally significant (0.72 [95% CI 0.53, 0.99]). The median OR for CPOE studies without CDS was 0.65 (IQR 0.13–0.72, 3 studies).

Duplicate errors and therapeutic duplications were the TGE most frequently identified [42, 43, 46, 52, 54, 70]. Selection errors, where prescribers mistakenly chose the wrong order from a drop-down menu or clicked the wrong item, were identified in four studies [40, 54, 56, 62]. A lack of advanced CDS was identified by the authors as a contributory factor to patient allergy errors [37, 39, 54]. Even where alert-based CDS was present, prescribing errors occurred due to prescribers overriding alerts [55] or making errors when typing dosages [54, 64]. Invalid orders were identified in two studies, due to prescribers generating orders under a nurse’s login [61] or leaving the electronic order unsigned [53]. Three of the CPOE studies did not identify any TGE [57, 58, 63]. This was likely due to the error detection methods used—one study used voluntary error reports alone as the error detection method [63], while the others examined gentamicin [58] and opioid prescribing errors only [57].

#### ePrescribing

**Fig. 4:**
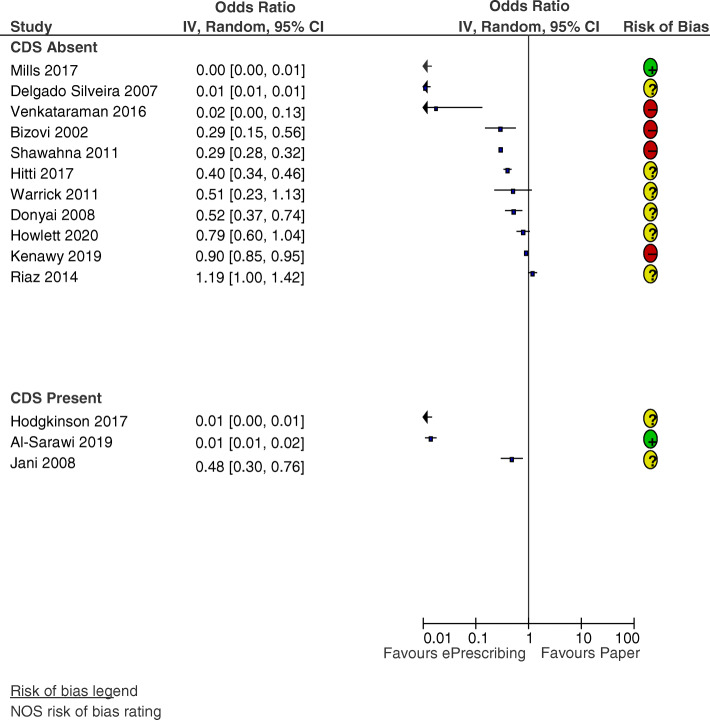
Forest plot of the odds ratio of prescribing errors for ePrescribing vs paper-based ordering, where CDS was absent (*n* = 11) or present (*n* = 3)

Eleven of the studies comparing the impact of ePrescribing with paper-based ordering were observed to have a lower OR of prescribing errors after the intervention (see Fig. 4). In three studies, the individual OR was not significant. Three commercial ePrescribing systems used alert-based CDS: Al-Sarawi et al. [38], Hodgkinson et al. [68], and Jani et al. [60] reported reductions in prescribing errors, with OR of 0.01 [95% CI 0.01–0.02], 0.01 [0.01–0.02], and 0.48 [0.30–0.76], respectively. The median OR for ePrescribing studies with CDS was 0.01 (IQR 0.01–0.48, 3 studies). The median OR for ePrescribing studies without CDS was 0.40 (IQR 0.0–0.79, 11 studies).

Selection errors [45, 49, 60, 68] and duplicate errors [38, 47, 60] were the TGE most frequently identified. Due to the simpler nature of some ePrescribing systems, free-text errors were possible [67]. Omission-based or prescription completeness errors were present due to the lack of forced fields in four studies [51, 66, 68, 69]. While Kenawy and Kett [69] reported the elimination of wrong-patient errors post-intervention, the simpler system employed by Venkataraman et al. [65] did not due to a reliance on manual entry of patient demographics by prescribers. Shawahna et al. [71] did not report TGE but acknowledged that the ePrescribing system did not reduce rates of dosing errors, due to a lack of CDS. Howlett et al. [59] calculated that 27% of errors detected post-implementation of EP were TGE.

### Effective BCTs in prescribing HIT implementation and optimisation

**Fig. 5:**
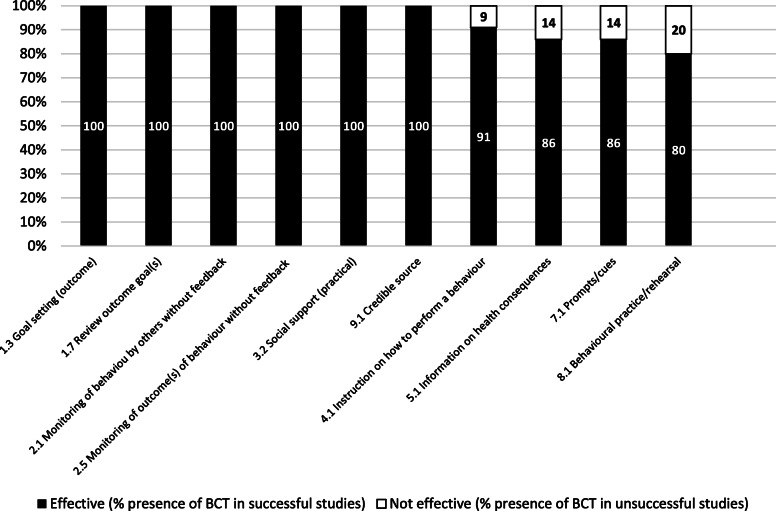
Stacked bar chart representing percentage effectiveness ratio [ER] of BCTs (*n* = 10), which is the number of times each BCT was coded in an effective intervention divided by the number of times it was coded in all studies included in the BCT analysis

After coding a sample of studies, it became apparent that the BCTs *12.1 Restructuring the physical environment* (coded due to the physical IT changes within the sites) and *4.1 Instruction on how to perform the behaviour* (coded due to the guided prescribing of CPOE/ePrescribing/CDS) were inherently present. Therefore, we chose to focus on additional BCTs, in order to identify those that had a causal effect on the success or otherwise of the interventions. BCTs that targeted prescribers’ behaviour were identified in 14 studies, with 18 individual BCTs identified. Twelve of the 14 studies demonstrated a reduction in the OR of prescribing errors and so were considered ‘successful’ interventions for the purpose of the BCTTv1 analysis. Two studies demonstrated insignificant results and so were considered ‘unsuccessful’. Ten BCTs were identified in two or more successful studies and so were considered effective. The ER was calculated for these 10 BCTs to interpret their effectiveness (see Fig. 5).

Six of the effective BCTs were unique to successful interventions (100% effectiveness ratios, or ER of 1): *1.3 Goal setting (outcome)*, *1.7 Review outcome goal(s)*, *2.1 Monitoring of behaviour by others without feedback*, *2.5 Monitoring of outcome(s) of behaviour without feedback*, *3.2 Social support (practical)*, and *9.1 Credible source.* The intervention components of these BCTs for HIT implementation included (i) prescriber or MDT involvement in system design, including prescribing functions and parameters; (ii) having clinical or IT colleagues present, or accessible by phone for practical guidance and to answer prescribers’ questions; and (iii) having a credible source such as a healthcare professional deliver any required training to clinical staff. Effective intervention components for continued HIT optimisation included (i) modification of the system in response to prescriber feedback, (ii) direct observation of prescriber workflow and behaviour in order to adapt a system and in turn modify prescriber behaviour, and (iii) monitoring of electronic prescriptions or orders generated by prescribers in order to prevent or detect errors.

Four BCTs were identified in both successful and unsuccessful interventions, and so lower weighted effectiveness ratios were determined. Two of these were specific to training methods: (i) didactic instruction on how to use the new system [ER 0.91] and (ii) practice prescribing sessions with a workbook or demonstration component [ER 0.8]. Two were related to the presence of CDS: (i) providing information on the consequences for the patient of prescribing a drug through CDS alerts [ER 0.86] and (ii) prompts or cues to the prescriber in the form of alerts or pop-ups, to encourage adjustment of an order [ER 0.86]. The resulting taxonomy of effective BCTs is presented in Table 2. Full-text excerpts used to code each BCT and the excluded BCTs are provided in Additional file 6.

**Table 2:** Effective behaviour change techniques to reduce prescribing errors in HIT

BCT cluster	BCT label	Key behaviour	ER (% effect. ratio)
**1. Goals and planning**	**1.3 Goal setting (outcome)**	✓ Ensure prescriber or clinical involvement in HIT configuration and design; in clinical parameter setting for dosing support and other clinical decision support; in drug library design	**1** (100)
	**1.7 Review outcome goal(s)**	✓ Review and modify HIT in response to prescriber feedback	**1** (100)
**2. Feedback and monitoring**	**2.1 Monitoring of behaviour by others without feedback**	✓ Observe and record prescriber workflow and behaviour with their knowledge but without providing feedback, in order to adapt system and in turn modify prescriber behaviour (e.g. drop-down menus that are contributing to selection errors may be modified after prescriber observation)	**1** (100)
	**2.5 Monitoring of outcome(s) of behaviour without feedback**	✓ Monitor electronic prescriptions or orders generated by prescribers without providing feedback in order to prevent or detect errors (not for the purpose of study data collection)	**1** (100)
**3. Social support**	**3.2 Social support (practical)**	✓ Ensure clinical colleagues (e.g. ‘super-users’) or IT phone support available to give practical system support to prescribers and to answer questions	**1** (100)
**9. Comparison of outcomes**	**9.1 Credible source**	✓ Deliver prescriber training, or information on the consequences of medication errors by a credible source such as an informatics pharmacist or other clinical healthcare professional	**1** (100)
**4. Shaping knowledge**	**4.1 Instruction on how to perform a behaviour**	✓ Provide training sessions on how to use the system and prescribe a drug correctly; may be classroom or workbook-based	**0.91** (91)
**5. Natural consequences**	**5.1 Information on health consequences**	✓ Alert the prescriber about the consequences of placing a specific medication order (e.g. patient allergy, drug-drug interaction, therapeutic duplication, contraindication) through system alerts or warnings; verbal or written information on medication errors may also be provided	**0.86** (86)
**7. Associations**	**7.1 Prompts/cues**	✓ Provide visual on-screen alerts or pop-ups to prompt prescribers to change or adjust potentially erroneous or unsafe medication orders	**0.86** (86)
**8. Repetition and substitution**	**8.1 Behavioural practice/rehearsal**	✓ Provide classroom or individual training sessions for prescribers to work through order examples, workbooks, online modules, or system demos	**0.80** (80)

#### Risk reduction and frequency of coded BCTs

While we primarily sought to identify the BCTs that facilitated the success of prescribing HIT, the number of unique BCTs coded in each study was also examined in order to determine whether a relationship existed between the frequency of BCTs and OR of prescribing errors. A greater median number of BCTs were observed to be coded in the successful studies (4 BCTs) versus the unsuccessful studies (2.5 BCTs). Similarly, a greater number of BCT clusters were identified in the studies that reported a decrease in prescribing errors (10 vs 4). The type of HIT did not affect the number of BCTs coded, as no substantial difference in BCTs was found in the CPOE (median 3, IQR 2–5.5) and ePrescribing (median 3, IQR 2.5–7) studies. Spearman’s correlation determined no association between the frequency of BCTs and OR of the intervention (*r*_*s*_ = − 0.049, *n* = 14, *p* = 0.868).

## Discussion

### Main findings

We reviewed 35 studies assessing the impact of HIT on prescribing errors. Both CPOE and ePrescribing interventions were examined in the review. The median OR for all studies comparing HIT with paper-based ordering was 0.24 (IQR 0.03–0.57, 35 studies). Individually, 28 studies were observed to have a lower OR of prescribing errors post-intervention. Seven studies demonstrated no significant reduction in OR. No substantial difference was observed between the success of CPOE and the success of ePrescribing. The presence of CDS in CPOE and ePrescribing systems was associated with a lower median OR of prescribing errors. Despite the absence of a meta-analysis, our summary of effect estimates agrees with the findings of previous systematic reviews on this topic, namely that prescribing HIT reduces the rates of prescribing errors in comparison to paper-based ordering [8, 9, 28, 74].

The novel aspect of the review was the construction of a tailored BCT taxonomy for the purpose of prescribing HIT design and implementation. Due to a lack of description of the interventions, the BCTTv1 analysis was limited to 14 of 35 studies. While no study explicitly stated the use of behavioural change theory in any part of their intervention, we were able to identify ten key BCTs across eight clusters that may influence prescribers’ behaviour.

The BCTTv1 analysis indicates that facilitators of success of prescribing HIT implementation and optimisation include ongoing user engagement and feedback, adequate troubleshooting support for prescribers, and medication error detection strategies, as the corresponding BCTs were identified only in successful studies. Classroom and workbook training and alert-based CDS to prompt prescribers while placing medication orders may also be effective in optimising prescribers’ behaviour. A correlation analysis revealed no association between the frequency of BCTs coded and the OR of prescribing errors, but a larger number of BCT clusters were coded in the successful studies.

### TGE—an ongoing issue

TGE or systems-related errors have been identified as unintended consequences of prescribing HIT since a seminal study by Koppel et al. [11] in 2005. TGE remain an issue despite the continued advancement of prescribing HIT [12, 75]. TGE identified in this review were duplicate orders, selection errors, errors related to a lack of CDS for patient allergy or overdose, and manual data input errors including one confirmed and one uncertain wrong-patient error. Drawing on the findings of the BCT analysis, user feedback, ongoing system assessment, and more robust methods of error detection may reduce the rates of TGE, regardless of the level of integrated CDS in a system.

TGE were absent from three of the studies in which BCTs were identified [57, 58, 71]. Two of these studies focused on medication errors in specific drug classes [57, 58]. The third study reported similar rates of dose errors pre- and post-implementation of their prescribing HIT, but no specific TGE [71]. It is likely that these findings are due to reporting constraints as opposed to a genuine absence of error.

### Implications for intervention design, implementation, and optimisation

Previous studies have identified the importance of good design and function in HIT; Han et al. [76] reported increased mortality rates in a paediatric ICU due to the unanticipated impact of CPOE on workflow. A pilot Delphi study of factors influencing the success and failure of HIT put forward that collaboration and goal setting within an organisation were contributors to successful HIT implementations, while a lack of understanding of the organisational context and changes to user workflow were potential failure criteria [77]. Debono et al. [24] identified key BCTs to address persistent environmental, social, and professional barriers experienced by nurses when using electronic medication management systems. However, HIT implementations in hospitals are not yet commonly guided by theory [78, 79]. Schwartzberg et al. ([80], p.109) went so far as to acknowledge that HIT is often designed in ‘a theoretical vacuum that will be subjected to unanticipated forces upon implementation’.

The findings of the BCTTv1 analysis have practical implications for design, implementation, and optimisation of prescribing HIT. A defined list of BCTs with key behaviours enables replication of our coding methods and may also be used as an evaluation tool. Successful BCTs may be targeted in future system implementations by using proposed strategies.

Prescribing medications is a complex cognitive task that may be composed of up to 30 subtasks [81]. Ideally, prescribers should be involved in system configuration as early as possible to ensure that workflows are functional. Order sets that do not match local guidelines [82] or ordering processes that encourage ‘workarounds’ such as the use of free-text fields to modify a prescription [83] are potential safety risks. While the prompts and cues provided by CDS contribute to safe prescribing, it is necessary to strike a balance with the frequency and manner of alerts that occur to avoid alert fatigue and potential adverse outcomes [84]. Involving prescribers in the configuration stage may result in the development of assistive, as opposed to interruptive or unnecessary alerts. It is also important to manage perceptions, as CDS does not replace clinical knowledge.

The observation of prescribers’ workflow for monitoring purposes was associated with successful interventions, as these observations in turn led to changes within the system to modify prescribers’ behaviour. An observation strategy may take the form of informal direct observation, a structured time and motion study, or using access logs that store timestamps when users perform specific actions on the system.

Monitoring of electronic prescriptions or orders as an outcome of prescriber behaviour was also associated with successful interventions. The volume of medication orders may increase after implementing CPOE, possibly due to prescribers’ unfamiliarity with the system [80]. Automated error detection tools, such as the validated Wrong-Patient Retract-and-Reorder too [85], or antidote-based trigger tools may be useful where a pharmacist review of every order is not possible.

Training programmes were more successful when delivered by a healthcare professional with knowledge of end-user workflow. Similarly, ‘super-users’ or healthcare professionals with additional training were a source of practical support for colleagues. Having one ‘super-user’ for two prescribers has been recommended for initial large-scale HIT implementations, with support gradually tapering off [86]. Additional roles for existing clinical staff, or the creation of clinical posts with responsibility for ongoing and new staff training and troubleshooting, are therefore key considerations for long-term success. The success of prescribing HIT is associated with a dynamic cycle of improvement and feedback, as opposed to a static and singular implementation. Formal and informal methods of feedback to and from users may be facilitated, such as feedback forms, medication safety huddles, and ‘lessons learned’ exchanges.

It is difficult to determine how intangibles like individual personalities, social capital of trainers, organisational culture, industrial relations, or luck contributed to the overall success of the individual interventions. BCTs focusing on nurses’ professional identity have previously been identified as contributary factors to the success of an electronic medication management system [24]. These intangibles and constructs were not reported in the included studies, so there is scope to build on our BCT taxonomy.

Recommendations for research include qualitative research focusing on users’ experiences of HIT, in order to add to the developing BCT taxonomy. Further studies of automated error detection methods and trigger tools in HIT systems may identify additional TGE and the prescriber behaviour that may lead to these types of errors.

### Limitations of the review

There were limitations to the review. Our quantitative synthesis presented a descriptive summary of a standardised metric in the form of odds ratios to determine the effectiveness of individual studies, as a meta-analysis was not appropriate. While our summarised findings are in agreement with previous reviews, conclusions of effectiveness of prescribing HIT should be interpreted with caution due to the heterogeneous nature of the included studies and frequent high risk of bias.

A BCT analysis was not possible for 21 of the included studies. It is likely that publication constraints led to missed details on potential BCTs that were carried out as part of implementation or optimisation in the included studies. While prescribing HIT research presents challenges to reporting, inclusion of contextual factors related to intervention design and delivery may be encouraged through the use of the Statement on Reporting of Evaluation Studies in Health Informatics (STARE-HI) checklist [87]. The BCT analysis itself involved interpretation of the BCTTv1 taxonomy in order to apply it to the interventions in the study. Furthermore, the BCTs that were excluded, or judged to be less effective, may prove effective in different contexts.

## Conclusions

Prescribing HIT is consistently associated with a reduction in prescribing errors, but as complex sociotechnical interventions, evaluation of contextual facilitators of success is important. By retrospectively applying the BCTTv1 to identify influences on prescribers’ behaviour, we have added a unique dimension of understanding to the body of work on prescribing HIT. The potentially effective BCTs identified in this review may be considered in the design of interventions, and as a reporting or evaluative tool. Developing and trialling new BCTs in the clusters identified within the tailored taxonomy may enhance the success of prescribing HIT implementations and contribute to long-term system optimisation.

## Supplementary Information

**Additional file 1.** Reporting guidelines. Description of data: Completed PRISMA and SWiM checklists.

**Additional file 2.** Search strings. Description of data: Systematic review search strings.

**Additional file 3.** BCT coding manual. Description of data: Coding manual constructed for BCT analysis.

**Additional file 4.** Supplementary information on included studies. Description of data: Table of included studies with detailed characteristics.

**Additional file 5.** Risk of bias assessment. Description of data: Risk of bias assessment including Newcastle-Ottawa Scale and funnel plots.

**Additional file 6.** Supplementary BCT files. Description of data: Table of BCTs with corresponding text extracted from the relevant study, table of BCTs excluded from the taxonomy, and correlational data.

## Data Availability

The datasets used and/or analysed during the current study are available from the corresponding author on reasonable request.

## Abbreviations

BCT: Behaviour change technique
BCTTv1: Behaviour change technique taxonomy version 1
CDS: Clinical decision support
CPOE: Computerised provider order entry
ER: Effectiveness ratio
HIT: Health information technology
IQR: Interquartile range
MN-CMS: Maternal and Newborn Clinical Management System
NOS: Newcastle-Ottawa Scale
OR: Odds ratio
