# Supplementary material for: The impact of health information technology on prescribing errors in hospitals: a systematic review and behaviour change technique analysis
Source: Syst Rev. 2020 Dec 3;9:275. doi: 10.1186/s13643-020-01510-7 (PMC7716445; doi:10.1186/s13643-020-01510-7)
Supplement: Supplementary file 2 — Additional file 2. Search strings. Description of data: Systematic review search strings. [file 13643_2020_1510_MOESM2_ESM.docx]

MEDLINE

(((((hospital[Title/Abstract] OR inpatient[Title/Abstract]) OR intensive care[Title/Abstract]) OR ("Hospitalization"[Mesh] OR "Critical Care"[Mesh])) AND ((((((((((("electronic prescribing"[MeSH Terms] OR ("electronic"[All Fields] AND "prescribing"[All Fields]) OR "electronic prescribing"[All Fields]) OR e-prescribing[Title/Abstract]) OR eprescribing[Title/Abstract]) OR computerised provider order entry[Title/Abstract]) OR computerized provider order entry[Title/Abstract]) OR CPOE[Title/Abstract]) OR EHR[Title/Abstract]) OR electronic health record[Title/Abstract]) OR electronic medical record[Title/Abstract]) OR EMR[Title/Abstract]) OR (((electronic[Title/Abstract] OR computerised[Title/Abstract]) OR computerized[Title/Abstract]) or barcode medication[Title/Abstract]) OR e-health[Title/Abstract]) OR e-prescribing[Title/Abstract]) OR eprescribing[Title/Abstract]) OR (("Medical Records Systems, Computerized"[Mesh] OR "Electronic Prescribing"[Mesh]) OR "Medical Informatics Applications"[Mesh]) AND (((((medication error[Title/Abstract] OR adverse drug event[Title/Abstract]) OR medication safety[Title/Abstract]) OR medication risk[Title/Abstract]) OR medication alert[Title/Abstract]) OR "Medication Errors"[Mesh])

CINAHL

(medication error OR adverse drug event OR medication safety OR medication risk OR medication alert OR ') AND (electronic prescribing OR eprescribing OR e-prescribing OR CPOE OR computerised provider order entry OR computerized provider order entry OR barcode medication OR electronic health record OR EHR OR EMR OR electronic medical record OR medical informatics applications) AND (hospital OR inpatient OR intensive care OR critical care)

EMBASE

('medication error':ab,ti OR 'adverse drug event':ab,ti OR 'medication safety':ab,ti OR 'medication risk':ab,ti OR 'medication alert':ab,ti OR 'medication error'/exp OR 'medication safety'/exp OR 'adverse drug reaction'/exp) AND ('electronic prescribing':ab,ti OR 'eprescribing':ab,ti OR 'e-prescribing':ab,ti OR 'CPOE':ab,ti OR 'computerised provider order entry':ab,ti OR 'computerized provider order entry':ab,ti OR 'electronic health record':ab,ti OR 'EHR':ab,ti OR 'EMR':ab,ti OR 'electronic medical record':ab,ti OR 'medical informatics applications':ab,ti OR 'electronic prescribing'/exp OR 'computerized provider order entry'/exp OR ‘barcode medication’:ab,ti OR 'electronic medical record'/exp OR 'medical informatics'/exp) AND ('hospital':ab,ti OR 'inpatient':ab,ti OR 'intensive care':ab,ti OR 'critical care':ab,ti OR 'hospital'/exp OR 'hospital patient'/exp OR 'intensive care'/exp)
