# Supplementary material for: The impact of health information technology on prescribing errors in hospitals: a systematic review and behaviour change technique analysis
Source: Syst Rev. 2020 Dec 3;9:275. doi: 10.1186/s13643-020-01510-7 (PMC7716445; doi:10.1186/s13643-020-01510-7)
Supplement: Supplementary file 3 — Additional file 3. BCT coding manual. Description of data: Coding manual constructed for BCT analysis. [file 13643_2020_1510_MOESM3_ESM.docx]

| BCT Coding Manual | | |
| --- | --- | --- |
| **Grouping of BCTs** | **Definition** | **Examples** |
| **1. Goals and planning** | | |
| **1.2 Problem solving** | Analyse, or prompt the person to analyse, factors influencing the behaviour and generate or select strategies that include overcoming barriers and/or increasing facilitators (includes ‘Relapse Prevention’ and ‘Coping Planning’).  Note: barrier identification without solutions is not sufficient. If the BCT does not include analysing the behavioural problem, consider 12.3,  Avoidance/changing exposure to cues for the behaviour, 12.1, Restructuring the physical environment, 12.2, Restructuring the social environment, or 11.2, Reduce negative emotions. | - MDT involvement in devising strategies to address conflict between system functionality, operational and safety priorities in order to achieve goal of optimisation of prescribing. |
| **1.3 Goal setting (outcome)** | Set or agree on a goal defined in terms of a positive outcome of wanted behaviour.  Note: only code guidelines if set as a goal in an intervention context; if goal is a behaviour, code 1.1, Goal setting (behaviour); if goal unspecified code 1.3, Goal setting (outcome). | - Prescriber/MDT involvement in system configuration, i.e. order set design, drug library construction, goal or parameter setting for CDS and dosing support. |
| **1.6 Discrepancy between current behaviour and goal** | Draw attention to discrepancies between a person’s current behaviour (in terms of the form, frequency, duration, or intensity of that behaviour) and the person’s previously set outcome goals, behavioural goals or action plans (goes beyond selfmonitoring of behaviour)  Note: if discomfort is created only code  13.3, Incompatible beliefs and not 1.6,  Discrepancy between current behaviour and goal; if goals are modified, also code 1.5, Review behaviour goal(s) and/or 1.7, Review outcome goal(s); if feedback is provided, also code 2.2, Feedback on behaviour. | - Training to mitigate errors related to specific user behaviours not compatible with optimisation of prescribing. |
| **1.7 Review outcome goal(s)** | Review outcome goal(s) jointly with the person and consider modifying goal(s) in light of achievement. This may lead to resetting the same goal, a small change in that goal or setting a new goal instead of, or in addition to the first.  Note: if goal specified in terms of  behaviour, code 1.5, Review behaviour goal(s), if goal unspecified, code 1.7, Review outcome goal(s); if discrepancy created consider also 1.6, Discrepancy between current behaviour and goal. | - Review and modify the system or intervention in response to prescriber feedback. |
|  |  |  |
| **2. Feedback and monitoring** | | |
| **2.1 Monitoring of behaviour by others without feedback** | Observe or record behaviour with the  person’s knowledge as part of a behaviour change strategy.  Note: if monitoring is part of a data  collection procedure rather than a  strategy aimed at changing behaviour, do not code; if feedback given, code only 2.2, Feedback on behaviour, and not 2.1, Monitoring of behaviour by others without feedback; if monitoring outcome(s) code 2.5, Monitoring outcome(s) of behaviour by others without feedback; if selfmonitoring behaviour, code 2.3, Selfmonitoring of behaviour. | - Observe or record prescriber workflow and behaviour with their knowledge but without providing feedback to the prescriber in order to adapt system and in turn modify prescriber behaviour, e.g. drop-down menus that are contributing to prescriber selection errors may be modified after a period of observation; observation of prescriber workflow by support staff during ‘Go-Live’ may be fed back to the main project team for the purpose of immediate system optimisation. |
| **2.2 Feedback on behaviour** | Monitor and provide informative or evaluative feedback on performance of the behaviour (e.g. form, frequency, duration, intensity).  Note: if Biofeedback, code only 2.6,  Biofeedback and not 2.2, Feedback on  behaviour; if feedback is on outcome(s) of behaviour, code 2.7, Feedback on  outcome(s) of behaviour; if there is no clear evidence that feedback was given, code 2.1, Monitoring of behaviour by others without feedback; if feedback on  behaviour is evaluative e.g. praise, also  code 10.4, Social reward. | - Feedback on errors related to a prescriber’s behaviour provided directly to the prescriber. |
| **2.5 Monitoring of outcome(s) of behaviour without feedback** | Observe or record outcomes of behaviour with the person’s knowledge as part of a behaviour change strategy.  Note: if monitoring is part of a data  collection procedure rather than a  strategy aimed at changing behaviour, do not code; if feedback given, code only 2.7, Feedback on outcome(s) of behaviour; if monitoring behaviour code 2.1, Monitoring of behaviour by others without feedback; if selfmonitoring outcome(s), code 2.4, Selfmonitoring of outcome(s) of behaviour. | - Monitor electronic prescriptions or orders generated by prescribers without providing feedback in order to detect and prevent errors (not for the purpose of study data collection). |
|  |  |  |
| **3. Social support** | | |
| **3.2 Social support (practical)** | Advise on, arrange, or provide practical  help (e.g. from friends, relatives, colleagues, ‘buddies’ or staff) for performance of the behaviour.  Note: if emotional, code 3.3, Social  support (emotional); if general or  unspecified, code 3.1, Social support  (unspecified) If only restructuring the  physical environment or adding objects to the environment, code 12.1, Restructuring the physical environment or 12.5, Adding objects to the environment; attending a group or class and/or mention of ‘follow up’ does not necessarily apply this BCT, support must be explicitly mentioned. | - Clinical colleagues or IT phone support available to give practical system support to prescribers or to answer questions, e.g. “super-users”. |
|  |  |  |
| **4. Shaping knowledge** | | |
| **4.1. Instruction on how to perform a behaviour** | Advise or agree on how to perform the behaviour (includes ‘Skills training’).  Note: when the person attends classes  such as exercise or cookery, code 4.1,  Instruction on how to perform the behaviour, 8.1, Behavioural practice/rehearsal and 6.1, Demonstration of the behaviour. | - Guided prescribing provided by CPOE/ePrescribing/CDS. - Training given to prescribers on how to use the system and how to prescribe a drug correctly. |
|  |  |  |
| **5. Natural consequences** | | |
| **5.1 Information on health consequences** | Provide information (e.g. written, verbal, visual) about health consequences of performing the behaviour.  Note: consequences can be for any target, not just the recipient(s) of the  intervention; emphasising importance of consequences is not sufficient; if  information about emotional  consequences, code 5.6, Information  about emotional consequences; if about social, environmental or unspecified consequences code 5.3, Information about social and environmental consequences. | - CDS that includes system alerts or warnings that inform the prescriber about the consequences of placing a specific medication order, e.g. patient allergy, drug-drug interaction, therapeutic duplication, contraindication. |
| **5.2 Salience of consequences** | Use methods specifically designed to  emphasise the consequences of  performing the behaviour with the aim of making them more memorable (goes  beyond informing about consequences).  Note: if information about consequences, also code 5.1, Information about health consequences, 5.6, Information about  emotional consequences or 5.3,  Information about social and environmental consequences. | - Educate prescribers on medication error scenarios and their consequences using specific examples and multimedia. |
|  |  |  |
| **6. Comparison of behaviour** | | |
| **6.1 Demonstration of the behaviour** | Provide an observable sample of the  performance of the behaviour, directly in person or indirectly e.g. via film, pictures, for the person to aspire to or imitate (includes ‘Modelling’).  Note: if advised to practice, also code, 8.1, Behavioural practice and rehearsal; If provided with instructions on how to perform, also code 4.1, Instruction on how to perform the behaviour. | - Provide prescribers with a demo version of the HIT for further training and practice. |
|  |  |  |
| **7. Associations** | | |
| **7.1 Prompts/cues** | Introduce or define environmental or  social stimulus with the purpose of  prompting or cueing the behaviour. The  prompt or cue would normally occur at  the time or place of performance.  Note: when a stimulus is linked to a  specific action in an if-then plan including one or more of frequency, duration or intensity also code 1.4, Action planning. | - CDS that includes on-screen alerts or pop-ups to prompt prescribers to change/adjust potentially erroneous or unsafe medication order. |
|  |  |  |
| **8. Repetition and substitution** | | |
| **8.1 Behavioural practice/rehearsal** | Prompt practice or rehearsal of the  performance of the behaviour one or more times in a context or at a time when the performance may not be necessary, in order to increase habit and skill.  Note: if aiming to associate performance with the context, also code 8.3, Habit formation. | - Classroom or individual training sessions where prescribers could work through prescribing examples, workbooks, online modules, or system demos. |
| **8.6 Generalisation of a target behaviour** | Advise to perform the wanted behaviour, which is already performed in a particular situation, in another situation. | - Dual-prescribing process (filling in identical paper and electronic orders) for the purpose of familiarising staff with the target behaviour. |
|  |  |  |
| **9. Comparison of outcomes** | | |
| **9.1 Credible source** | Present verbal or visual communication  from a credible source in favour of or against the behaviour.  Note: code this BCT if source generally  agreed on as credible e.g., health  professionals, celebrities or words used to indicate expertise or leader in field and if the communication has the aim of persuading; if information about health consequences, also code 5.1, Information about health consequences, if about emotional consequences, also code 5.6, Information about emotional  consequences; if about social,  environmental or unspecified consequences also code 5.3, information about social and environmental consequences. | - Prescriber training, or information on the consequences of medication errors delivered by a credible source e.g. informatics pharmacist, other clinical healthcare professional, peer to peer learning within own discipline. |
| **9.2 Pros and cons** | Advise the person to identify and compare reasons for wanting (pros) and not wanting to (cons) change the behaviour (includes ‘Decisional balance’).  Note: if providing information about  health consequences, also code 5.1,  Information about health consequences; if providing information about emotional consequences, also code 5.6, Information about emotional consequences; if providing information about social, environmental or unspecified consequences also code 5.3, Information about social and environmental consequences. | - Request prescribers to identify the advantages and disadvantages of the new technology in comparison to the previous paper ordering system. |
|  |  |  |
| **12. Antecedents** | | |
| **12.1 Restructuring the physical environment** | Change, or advise to change the physical environment in order to facilitate performance of the wanted behaviour or create barriers to the unwanted behaviour (other than prompts/cues, rewards and  punishments).  Note: this may also involve 12.3,  Avoidance/reducing exposure to cues for the behaviour; if restructuring of the social environment code 12.2, Restructuring the social environment;  if only adding objects to the environment, code 12.5, Adding objects to the environment. | - Physical change from paper charts to electronic charts/CPOE/ePrescribing. Includes addition of computers on wheels and shared terminals. |
| **12.5 Adding objects to the environment** | Add objects to the environment in order to facilitate performance of the behaviour.  Note: Provision of information (e.g. written, verbal, visual) in a booklet or leaflet is insufficient. If this is accompanied by social support, also code 3.2, Social support (practical); if the environment is changed beyond the addition of objects, also code 12.1, Restructuring the physical environment | - Providing prescribers with individual technology to use for prescribing purposes, in addition to shared terminals, e.g. personal tablet device. |
