# Supplementary material for: The impact of health information technology on prescribing errors in hospitals: a systematic review and behaviour change technique analysis
Source: Syst Rev. 2020 Dec 3;9:275. doi: 10.1186/s13643-020-01510-7 (PMC7716445; doi:10.1186/s13643-020-01510-7)
Supplement: Supplementary file 4 — Additional file 4. Supplementary information on included studies. Description of data: Table of included studies with detailed characteristics. [file 13643_2020_1510_MOESM4_ESM.docx]

| Characteristics and summary of findings of the included studies | | | | | | | | | | | | |
| --- | --- | --- | --- | --- | --- | --- | --- | --- | --- | --- | --- | --- |
| **Study (Country)** | **Population** | **Prescribing-associated HIT** | **HIT detail** | **System** | **Design** | **No.**  **sites** | **Setting** | **Sample** | **Error detection method** | **Prescribing error rate**  **pre-intervention/ control** | **Prescribing error rate post-intervention** | **Technology-generated errors** |
| Abbass 2011 (USA) | Adult | CPOE | **Content** CPOE with order sets. | Commercial | Control group (retrospective) | 1 | All areas | **1110 orders** (Control: 563, Intervention: 547) | Chart review | 117/563 (20.8%) | 18/547 (3.3%) | Lack of CDS led to allergy/DDI errors. |
| Ali 2010 (UK) | Adult | CPOE | **Content** EHR; CPOE with order sets and allergy alerting. **Delivery** Interface was designed by MDT to enable ongoing customisation. | Commercial | Time series analysis (prospective) | 1 | ICU | **14721 prescriptions** (Pre: 4463, Post-1: 5083, Post-2: 5175) | Routine pharmacist review; Chart review | 745/4463 (16.7%) | 0/5175 | Allergy alert did not fire if the allergy field was not already completed by the prescriber. |
| Al-Sarawi 2019 (Australia) | Adult | EP | **Content** EHR and EP. | Commercial | Pre-post (prospective) | 3 | All areas | **4689 orders** (Pre: 1145, Post: 3544) | Chart review | 776/1145 (67.7%) | 101/3544 (2.8%) | Duplicate orders increased post-CPOE. |
| Armada 2014 (Spain) | Adult | CPOE | **Content** CPOE with dosing support and allergy, DDI, and duplicate alerting; integrated local treatment protocols. **Delivery** 8-hour computer training programme for users. | Commercial | Time series analysis (prospective) | 1 | ICU | **5729 orders** (Pre: 1829, Post-1: 1806, Post-2: 2094) | Routine pharmacist review | 819/1829 (44.8%) | 16/2094 (0.8%) | Selection errors made while searching for drugs on drop-down menus. |
| Bates 1998 (USA) | Adult | CPOE | **Content** EHR; CPOE with menu of medications and potential doses; limited DDI, allergy and lab checking. | Homegrown | Pre-post (prospective) | 1 | 2 medical wards; 2 surgical wards; 2 ICUs | **24453 patient days** (Pre: 12218, Post: 11235) | Routine pharmacist review; Chart review | 611/12218 (50/1000 pt. days) | 326/11235 (29/1000 pt. days) | Increase in therapeutic duplication of sedating drugs, which the CPOE did not prevent. |
| Bates 1999 (USA) | Adult | CPOE | **Content** EHR; CPOE with allergy and DDI checking, and potassium ordering support. Medication administration record paper-based. | Homegrown | Time series analysis (prospective) | 1 | 3 medical units | **7985 patient days** (Pre: 1704, Post-1: 2619, Post-2: 1784, Post-3: 1878) | Routine pharmacist review; Chart review; Medication order review | 168/10070 (98.6/1000 pt. days) | 48/14352 (25.5/1000 pt. days) | Missed dose errors (not main outcome of interest) increased with CPOE. |
| Bizovi 2002 (USA) | Adult/  Paediatric | EP | **Content** EHR with EP pick-list; weight-based dosing for those weighing <50kg. Free text fill-in prescriptions were available.  **Delivery** Training hand-out for prescribers. | Commercial | Pre-post (retrospective) | 1 | ED | **3920 prescriptions**  (Pre: 2326,  Post: 1594) | Routine pharmacist review;  Medication order review | 54/2326 (2.32%) | 11/1594 (0.69%) | Free-text electronic prescriptions had a higher rate of error than the pick-list prescriptions. |
| Boling 2005 (USA) | Paediatric | CPOE | **Content** EHR; CPOE with paediatric age and weight-based dose range checking, dosing alerts. | Commercial | Pre-post (prospective) | 1 | All areas | **21253 orders** (Pre: 13997,  Post: 7256) | Trigger tool methodology; Chart review; Voluntary error reports | Opioid prescribing errors 8/13997 (0.57/1000 doses) | 1/7256 (0.14/1000 doses) | None found. |
| Colpaert 2006  (Belgium) | Adult | CPOE | **Content** EHR; CPOE with CDS incorporating specific patient prescription protocols (e.g. liver transplant PTs), DDI and allergy alerts. Drop-down menus avoided to prevent selection errors. | Commercial | Prospective controlled trial | 1 | 3 units in an ICU | **2510 prescriptions**  (Intervention: 1286,  Control: 1224) | Chart review; Medication order review | 331/1224 (27.0%) | 44/1286 (3.4%) | CPOE errors were mostly duplicate prescriptions. |
| Cordero 2004  (USA) | Neonatal | CPOE | **Content** EHR; CPOE with NICU-specific order sets, allergy checking, dose checking, and weight-based prescribing.  **Delivery** ‘Super-users’ received 16 hours training, prescribers 2-4 hours. 24-hour IT support during implementation. | Commercial | Pre-post (retrospective) | 1 | NICU | **211 patients**  (Pre: 111, Post:100 post) | Chart review | Gentamicin prescribing errors 14/111 (12.6%) | 0/100 | None found. |
| Delgado Silveira 2007  (Spain) | Adult | EP | **Content** EP form with manual access to hospital formulary and guidelines; no inbuilt CDS. | Commercial | Pre-post (prospective) | 1 | 2 medical units | **4814 prescriptions**  (Pre: 2848,  Post: 1966) | Routine pharmacist review | 2683/2848 (94.2%) | 299/1966 (15.2%) | Drug interaction errors increased with CPOE, this was not significant. |
| Donyai 2008 (UK) | Adult | EP | **Content** EHR; EP with formulary and dose guidelines; closed-loop administration system. | Commercial | Pre-post (prospective) | 1 | Surgical ward | **4803 orders**  (Pre: 2450,  Post: 2353) | Routine pharmacist review;  Chart review;  Medication order review | 94/2450 (3.8%) | 48/2353 (2.0%) | Selection errors were found post-EP. 1 wrong-patient error post-EP, authors uncertain if TGE. |
| Hernandez 2015  (France) | Adult | CPOE | **Content** CPOE with order sets, drug selection menus, and CDS for allergy checking, therapeutic duplications, DDIs, and dose and age range checking. | Commercial | Pre-post (prospective) | 1 | Orthopaedic unit | **2981 orders** (Pre: 1593,  Post: 1388)  **2635 OFEs**  (Pre: 1222,  Post: 1407 | Chart review; Direct observation | 479/1593 (30.1%) | 33/1388 (2.4%) | Duplicate orders increased with CPOE. |
| Hitti 2017 (Lebanon) | Adult | EP | **Content** Discharge EP which included 166 commonly prescribed drugs and dosing functions. A free-text option was available to prescribers. Weight-based dosing for paediatrics, manual calculation was required. | Homegrown | Pre-post (prospective) | 1 | ED | **2883 prescriptions**  (Pre: 1475,  Post: 1408) | Chart review | 999/1475 (67.7%) | 641/1408 (45.5%) | Duplicate errors increased with CPOE. |
| Hodgkinson 2017  (Australia) | Adult/  Paediatric | EP | **Content** EP with CDS for allergy, DDI, therapeutic duplicates, and paediatric weight-based dosing.  **Delivery** Training delivered by a pharmacist involving presentations, tutorials, and an online module. Ad-hoc training as needed. | Commercial | Pre-post (prospective) | 1 | ED and OPD | **1289 orders**  (Pre: 654,  Post: 635) | Routine pharmacist review; Medication order review | 360/379 (95.0%) | 51/375 (13.6%) | 50 systems-related errors post CPOE, such as selection errors or not filling in necessary fields. |
| Howlett 2020  (Ireland) | Paediatric | EP | **Content** EP with standardised concentration infusions replacing weight-based infusions; smart pumps. Dual paper/EP system for 4 months.  **Delivery** Information and training sessions were held in the weeks before implementation. Prescriber training was mandatory. | Commercial | Time series analysis (retrospective) | 1 | PCCU | **3356 orders**  (Pre: 1202,  Post-1: 246,  Post-2: 752,  Post-3: 1156) | Routine pharmacist review | 123/1020 (10.2%) | 113/1156 (9.8) | Incorrect formulation and dose errors increased with CPOE. |
| Jani 2008  (UK) | Paediatric | EP | **Content** EP linked to patient management system; no EHR. Drugs are selected from a menu. Basic CDS alerts for weight/height outside expected age range, for allergy, and exact drug duplicates. | Commercial | Pre-post (retrospective) | 1 | Nephrology OPD | **2222 orders**  (Pre: 1574,  Post: 648) | Routine pharmacist review;  Chart review | 112/1574 (7.1%) | 23/648 (3.5%) | Duplicate orders increased with CPOE. Wrong route, frequency and overdose also found due to selection errors. |
| Kadmon 2009  (Israel) | Paediatric | CPOE | **Content** EHR; CPOE with weight-based dosing alerts. No allergy or DDI alerting. The computer did not accept any orders over the legal dosing limit. | Commercial | Time series analysis (retrospective) | 1 | PICU | **5000 orders**  (Pre: 1250,  Post-1: 1250,  Post-2: 1250,  Post-3: 1250) | Medication order review | 69/1250 (5.5%) | 9/1250 (0.7%) | Prescriptions were found to be prescribed by nurses, due to doctors using computers where a nurse was already logged in. |
| Kazemi 2011 (Iran) | Neonatal | CPOE | **Content** EHR; CPOE and EMAR with drop-down lists and order sets. CDS adjusts dose for diagnosis, age, weight and glomerular filtration rate. The prescriber can accept or ignore the dose correction.  **Delivery** Group and individual training for prescribers, demo also available. | Commercial | Time series analysis (prospective) | 1 | Neonatal unit | **4508 medication-days**  (Pre: 1688,  Post-1: 1489,  Post-2: 1331) | Medication order review | 876/1688 (51.9%) | 442/1331 (33.2%) | "Neighbouring cell" errors were noted, where a prescriber chose a nearby cell in error or used incorrect data to do dose calculations. |
| Kenawy 2019  (Egypt) | Adult/  Paediatric | EP | **Content** EP with drop-down menus for selecting drugs and dosages. Patient details were entered manually. No CDS. | Commercial | Pre-post (retrospective) | 1 | 4 OPDs (Cardiology, nephrology, paediatric, neurology) | **25057 orders**  (Pre: 12249,  Post: 11808) | Voluntary error reports | 3467/12249 (28.3%) | 3100/11808 (26.3%) | Indication and omission prescribing errors increased with CPOE. |
| King 2003  (Canada) | Paediatric | CPOE | **Content** CPOE integrated with lab, no CDS. | Commercial | Pre-post (retrospective) | 1 | 3 medical wards; 2 surgical wards | **12460 patients**  (Pre: 6674,  Post: 5786) | Voluntary error reports | 7/6674 (0.10%) | 4/5786 (0.07%) | None found. |
| Liao 2017  (USA) | Adult | CPOE | **Content** EHR; CPOE with pharmacy order verification, order sets and pre-built orders.  **Delivery** 4-hour training session for prescribers. ‘Super-users’ available for first 3 weeks of implementation. | Commercial | Time series analysis (prospective) | 1 | ICU | **3988 patient days**  (Pre: 888,  Post-1: 1103,  Post-2: 937,  Post-4: 1060) | Chart review | 769/888 (0.86/ pt. day) | 327/1060 (0.31/ pt. day) | Reduction in errors only evident 2 years post-implementation. |
| Mahoney 2007  (USA) | Adult/  Paediatric | CPOE | **Content** EHR; CPOE and CDS with allergy, duplicate, and dose alerts; case-specific order sets; alerts for drug monitoring. **Delivery** MDT team involvement with build. Classroom, online and workbook training for users. | Commercial | Pre-post (retrospective) | 3 | All areas | **2843165 orders**  (Pre: 1452346, Post: 1390789) | Routine pharmacist review | 4815/1452346 (0.33%) | 2227/1390789 | Duplicate errors did not significantly decrease with CPOE. |
| Mills 2017  (UK) | Adult | EP | **Content** Discharge EP with medications selected from a menu of those prescribed as an inpatient, new ones may be added. | Commercial | Pre-post (retrospective) | 1 | All areas | **318 patients**  (Pre: 159, Post: 159) | Chart review; Medication order review | 158/159 (99.4%) | 37/159 (23.3%) | 8/37 errors post-CPOE were selection errors on menus. |
| Pontefract 2018  (UK) | Adult | CPOE | **Content** CPOE and CDS (similar levels in the 3 sites) for 78 high-alert medications. | Commercial | Pre-post (prospective) | 3 | All areas | **2422 patients**  (Pre: 1244, Post: 1178) | Trigger tool methodology; Routine pharmacist review;  Chart review | 562/11312 (5.0%) | 390/9826 (4.0%) | Increase in insulin prescribing errors with CPOE in 1 site due to lack of CDS. |
| Potts 2004  (USA) | Paediatric | CPOE | **Content** EHR and CPOE with CDS for allergy, dose checking, DDI alerts, renal dose adjustment and US Food and Drug alerts. >900 order sets.  **Delivery** Training for users 1 month before implementation. | Homegrown | Pre-post (prospective) | 1 | CCU | **13828 orders**  (Pre: 6803,  Post: 7025) | Routine pharmacist review; Medication order review | 2049/6803 (30.1%) | 12/7025 (0.2%) | Dose errors related to trailing decimal points or missing weights occurred with CPOE. |
| Riaz 2014  (Pakistan) | Adult | EP | **Content** EP system. Free-text fields available. | Homegrown | Control group (prospective) | 2 | 2 OPD and 2 ED | **2040 prescriptions**  (Control: 1020,  Intervention: 1020) | Medication order review | 530/1020 (52.0%) | 574/1020 (56.3%) | Omission errors higher on CPOE prescriptions which caused error increase. |
| Rouayroux 2019  (France) | Adult | CPOE | **Content** CPOE with allergy and contraindication alerts. | Commercial | Time series analysis (retrospective) | 1 | Cardiology and diabetes depts. | **3086 patient days**  (Pre: 1001,  Post-1: 1016,  Post-2: 1069) | Routine pharmacist review | 121/1001 (12.1%) | 105/1069 (9.8%) | Unit of use errors and duplicate orders increased with CPOE. |
| Shawahna 2011  (Pakistan) | Adult/  Paediatric | EP | **Content** EP on handheld computers with tick boxes indicating dosing instructions, frequency and route. No CDS. Linked to a medication formulary.  **Delivery** 1-day training workshop for prescribers. Seminars and newsletters also provided. | Homegrown | Pre-post (prospective) | 1 | All areas | **32662 orders** (Pre: 15808,  Post: 16854) | Chart review; Medication order review | 3426/15808 (21.7%) | 1270/16854 (7.5%) | None found. |
| Shulman 2005  (UK) | Adult | CPOE | **Content** CPOE with access to a local formulary and guidelines. No CDS.  **Delivery** Staff training provided, system changeover happened in one day. | Commercial | Pre-post (prospective) | 1 | ICU | **3465 prescriptions**  (Pre: 1036, Post: 2429) | Routine pharmacist review | 66/1036 (6.4%) | 114/2429 (4.7%) | Errors related to overdose increased with CPOE, with the potential to cause serious morbidity or mortality. Orders were frequently unsigned and therefore invalid. |
| Spencer 2005  (USA) | Adult | CPOE | **Content** CPOE with allergy alerts and duplicate order screening. | Commercial | Pre-post (prospective) | 1 | 2 medical units | **4339 patient discharges**  (Pre: 2714,  Post: 1625) | Voluntary error reports | 38/2714 (0.014 prescribing errors/pt. discharge) | 13/1625 (0.008 prescribing error/pt. discharge] | 23 reported errors caused by CPOE, including allergy errors, duplicate orders, input errors and discrepancies when transcribing to pharmacy computer. |
| Van Doormaal 2009  (The Netherlands) | Adult | CPOE | **Content** CPOE and CDS with order sets and protocols, allergy alerts, DDI alerts and overdosing alerts. | Commercial (Site 1)  Homegrown  (Site 2) | Pre-post (prospective) | 2 | 4 medical wards | **1195 patients**  (Pre: 592,  Post: 603) | Chart review; Medication order review | 5724/7286 (78.6%) | 1355/7058 (19.2%) | Overriding of alerts occurred with CPOE due to alert fatigue. |
| Venkataraman 2016  (UK) | Paediatric | EP | **Content** EP for infusions with dosing calculations based on the recorded weight and age of the child. No CDS, not linked to an EHR. | Homegrown | Pre-post (prospective) | 1 | PCCU | **251 prescriptions**  (Pre: 132,  Post: 119 post) | Routine pharmacist review | 43/132 (32.6%) | 1/119 (0.8%) | Wrong-patient error due to manual input of date of birth. |
| Warrick 2011  (UK) | Paediatric | EP | **Content** EHR and EP with formulary, weight-based calculations, and overdue alerts. No CDS. | Commercial | Time series analysis (prospective) | 1 | PICU | **624 prescriptions**  (Pre: 159,  Post-1: 208,  Post-2: 257) | Chart review | 14/159 (8.8%) | 12/257 (4.6%) | Infusions were prescribed with no diluent or rate with CPOE. |
| Westbrook 2012  (Australia) | Adult | CPOE | **Content** EHR and CPOE with order sets, DDI alerts, and allergy alerts. | Commercial | Difference in differences (prospective) | 2 | 6 medical wards | **15194 patient days**  (Pre: 8805,  Post: 6389) | Routine pharmacist review | 4270/8805 (48.5/100 patient days) | 1029/6389 (16.1/100 patient days | Selection errors occurred with CPOE. |
