# Supplementary material for: The impact of health information technology on prescribing errors in hospitals: a systematic review and behaviour change technique analysis
Source: Syst Rev. 2020 Dec 3;9:275. doi: 10.1186/s13643-020-01510-7 (PMC7716445; doi:10.1186/s13643-020-01510-7)
Supplement: Supplementary file 5 — Additional file 5. Risk of bias assessment. Description of data: Risk of bias assessment including Newcastle-Ottawa Scale and funnel plots. [file 13643_2020_1510_MOESM5_ESM.docx]

| Newcastle-Ottawa Scale for assessing the quality of nonrandomized studies | | | | | | | |
| --- | --- | --- | --- | --- | --- | --- | --- |
| Study (year) | **Selection Criteria** | | **Comparability Criteria ^c^** | **Outcome Criteria** | | | **Risk of Bias Rating** |
|  | Representativeness of exposed cohort ^a^ | Cohort selection ^b^ |  | Blinding ^d^ | Adequate study prescribing errors period ^e^ | Adequate sample ^f^ |  |
| Abbass et al. (2011) | ** |  | ** |  | * | * | Medium |
| Ali et al. (2010) | ** |  | * |  | * | * | Medium |
| Al-Sarawi et al. (2019) | ** | * | * |  | * | * | Low |
| Armada et al. (2014) |  |  | * |  | * | * | High |
| Bates et al. (1998) | ** |  | ** |  | * | * | Medium |
| Bates et al. (1999) | ** |  | * |  | * | * | Medium |
| Bizovi et al. (2002) | ** |  | ** |  |  | * | High |
| Boling et al. (2005) |  | * | * |  |  | * | High |
| Colpaert et al. (2006) | ** |  | * | * | * | * | Medium |
| Cordero et al. (2004) |  |  | * |  |  | * | High |
| Delgado Silveira et al. (2007) | ** |  | * | * |  | * | Medium |
| Donyai et al. (2008) | ** |  | * |  | * | * | Medium |
| Hernandez et al. (2015) | ** |  | * | * | * | * | Medium |
| Hitti et al. (2017) | ** |  | ** | * |  | * | Medium |
| Hodgkinson et al. (2017) | ** |  | * |  | * | * | Medium |
| Howlett et al. (2020) | ** |  | * |  | * | * | Medium |
| Jani et al. (2008) | ** |  | * | * | * | * | Medium |
| Kadmon et al. (2009) | * |  | * |  | * | * | High |
| Kazemi et al. (2009) |  |  | * |  |  | * | High |
| Kenawy and Kett (2019) |  |  | * |  |  | * | High |
| King et al. (2003) |  |  | * |  | * | * | High |
| Liao et al. (2017) | ** |  | ** |  | * | * | Medium |
| Mahoney et al. (2007) |  | * | * |  | * | * | High |
| Mills et al. (2017) | ** | * | * |  | * | * | Low |
| Pontefract et al. (2018) |  |  | ** |  | * | * | High |
| Potts et al. (2003) | ** |  | * |  |  | * | High |
| Riaz et al. (2014) | ** |  | * |  | * | * | Medium |
| Rouayroux et al. (2019) | ** |  | * |  | * | * | Medium |
| Schawahna et al. (2010) | ** | * | * |  |  | * | High |
| Shulman et al. (2005) | ** |  | * | * | * | * | Medium |
| Spencer et al. (2005) |  | * | * |  | * | * | High |
| Van Doormal et al. (2009) | ** |  | ** |  |  | * | Medium |
| Venkataraman et al. (2016) | ** |  | * |  |  |  | High |
| Warrick et al. (2011) | ** |  | * |  | * | * | Medium |
| Westbrook et al. (2012) | ** |  | * |  | * | * | Medium |
| a Researchers reviewed data from all patients or charts or a randomised selection of all patients or charts (two stars), or included a randomised selection of charts or patients (one star);  b Sample includes all hospital patients (one star)  c Study included clear methods with multiple trained reviewers or methods to detect prescribing errors (one star) and controls for confounders (two stars);  d Reviewers of medication error were independent from the patient data under review and hospital staff were unaware of the study (one star);  e Data collection prescribing period was reported and allowed for a minimum 6-month shakedown prescribing period (one star);  f study included an adequate sample (at least 1000 patient-days, 100 patients, or 300 orders) (one star) | | | | | | | |
| Good quality: 3 stars in selection domain AND 1 or 2 stars in comparability domain AND 2 or 3 stars in outcome/exposure domain  Fair quality: 2 stars in selection domain AND 1 or 2 stars in comparability domain AND 2 or 3 stars in outcome/exposure domain  Poor quality: 0 or 1 star in selection domain OR 0 stars in comparability domain OR 0 or 1 stars in outcome/exposure domain | | | | | | | |


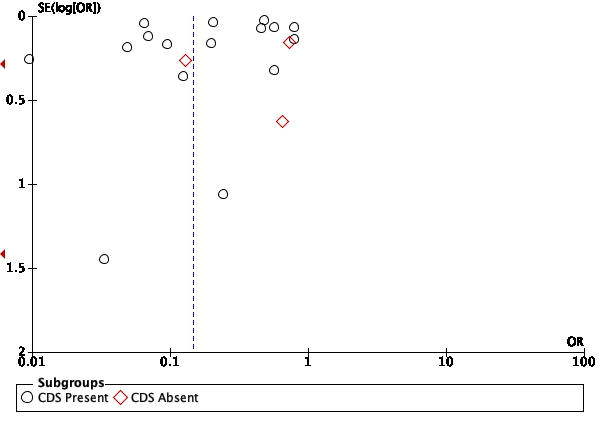

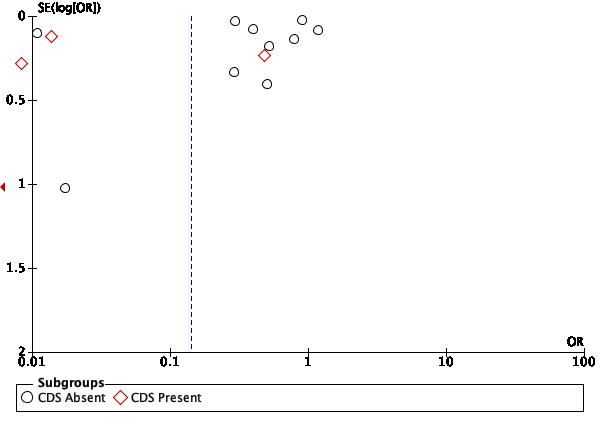


Figure AF5.1: Funnel plot of comparison CPOE vs Paper Figure AF5.2: Funnel plot of comparison ePrescribing vs Paper
