# Supplementary material for: The impact of health information technology on prescribing errors in hospitals: a systematic review and behaviour change technique analysis
Source: Syst Rev. 2020 Dec 3;9:275. doi: 10.1186/s13643-020-01510-7 (PMC7716445; doi:10.1186/s13643-020-01510-7)
Supplement: Supplementary file 6 — Additional file 6. Supplementary BCT files. Description of data: Table of BCTs with corresponding text extracted from the relevant study, table of BCTs excluded from the taxonomy, and correlational data. [file 13643_2020_1510_MOESM6_ESM.docx]

| Table AF6.1 Effective BCTs in prescribing HIT implementation and optimisation | | | | |
| --- | --- | --- | --- | --- |
| BCT cluster | BCT label | Key behaviour | Study [excerpt from text] | ER  (% effect.  Ratio) |
| 1. Goals and planning | 1.3 Goal setting (outcome) | - Ensure prescriber or clinical involvement in HIT configuration and design; in clinical parameter setting for dosing support and other clinical decision support; in drug library design | **Cordero 2004 –** [The NICU-specific physician order sets were developed on evidence provided by best practices by a subcommittee that met regularly and is composed of information system specialists, neonatal pharmacist, staff nurses, nurse managers, attending neonatologists, and prescribing paediatric chief residents. After development, six admission or procedural-type order sets and 12 medication decision support sets designed specifically for neonatal use were approved by a hospital-wide multidisciplinary committee that oversees CPOE activities of different units]  **Kazemi 2011 –** [Three neonatal sub-specialists reviewed the knowledge-base and approved its compliance with the original guidelines. A computer function was developed to calculate GFR for neonates based on the patient’s creatinine clearance, body surface area (BSA), age, and gestational age. A paediatric nephrologist tested the functionality of the GFR calculator, reviewed its compliance with the references, and approved it]  **Mahoney 2007 –** [each hospital established a physician-led multidisciplinary team to identify operational workflow changes and develop related policies and procedures. This multidisciplinary team also coordinated the implementation schedule]  **Venkataraman 2016 –** [The options of medications to be included, dilution method, and dosage range were agreed between PCCU consultants, pharmacy and nursing staff] | **1**  (100) |
|  | 1.7 Review outcome goal(s) | - Review and modify HIT in response to prescriber feedback | **Ali 2010 –** [The software allows the clinical design team to make changes ‘on the fly’ and to react to staff feedback, so that the interface is constantly evolving]  **Armada 2014 –** [The system has been refined through user feedback]  **Mahoney 2007 –** [clinicians have expressed improvement in the ordering of corollary medication orders and improved adherence to standards of care]  **Shawahna 2011 –** [Suggestions rendered by the collective were considered and the electronic inpatient record was modiﬁed accordingly, later, the ﬁnal design was sent for approval to a total of 200 doctors, 200 nurses and 100 pharmacists, employed by the hospital or similar settings in Lahore] | **1**  (100) |
| 2. Feedback and monitoring | 2.1 Monitoring of behaviour by others without feedback | - Observe and record prescriber workflow and behaviour with their knowledge but without providing feedback, in order to adapt system and in turn modify prescriber behaviour (e.g. drop-down menus that are contributing to selection errors may be modified after prescriber observation) | Ali 2010 – [Working practices were observed and recorded to ensure a smooth migration from paper to electronic patient record]  **Kazemi 2011 –** [In this period, when a resident complained about a warning that was perceived by him /her as being inappropriate, A.K. asked him/her to explain how did he/she calculate the dose and frequency for that medication] | **1**  (100) |
|  | 2.5 Monitoring of outcome(s) of behaviour without feedback | - Monitor electronic prescriptions or orders generated by prescribers without providing feedback in order to prevent or detect errors (not for the purpose of study data collection) | **Ali 2010 –** [Most prescriptions on admission are written by the surgical trainee handing the patient over, and are subsequently reviewed by the ICU staff]  **Shulman 2005 –** [In this study the pharmacist attended the ward round throughout the study], [The fact that these MEs were rectified before they harmed the patient underlines the value of daily prescription review by an experienced clinical pharmacist] | **1**  (100) |
| 3. Social support | 3.2 Social support (practical) | - Ensure clinical colleagues (e.g. ‘super-users’) or IT phone support available to give practical system support to prescribers and to answer questions | **Cordero 2004 –** [During the implementation of CPOE there was 24-hour support provided by information systems staff], [Nursing leaders identified to provide support for clinicians received 16 hours of classroom training]  **Hodgkinson 2017 –** [If required, ad hoc training was provided by an e-prescribing pharmacist and/or the outpatient clinic/ED pharmacist to manage any system-related errors identiﬁed]  **Liao 2017 –** [For 3 weeks during the implementation process, “super-users” were available in all clinical areas at all times] | **1**  (100) |
| 9. Comparison of outcomes | 9.1 Credible source | - Deliver prescriber training, or information on the consequences of medication errors by a credible source such as an informatics pharmacist or other clinical healthcare professional | **Hodgkinson 2017 –** [After-study clinicians received additional training on e-prescribing provided by an e-prescribing pharmacist]  **Kazemi 2011 –** [Two of the authors (A.K. and A.A.) conducted group and private training sessions for the residents to teach them how to use the prescription system]  **Venkataraman 2016 –** [All prescribers were formally briefed and trained for using EIC by the PCCU pharmacists] | **1**  (100) |
| 4. Shaping knowledge | 4.1 Instruction on how to perform a behaviour | - Provide training sessions on how to use the system and prescribe a drug correctly; may be classroom or workbook-based | **Armada 2014 –** [The staff followed a training program on the management of the computer system for 8 hours]  **Bizovi 2002 –** [Training in the use of the CA prescription writing included the production of a two-page handout that showed the steps needed to write CA prescriptions]  **Cordero 2004 –** [Formalized training for all NICU staff and physicians began 4 weeks prior to implementation] [Every physician received 2 to 4 hours of individualized instruction prior to the initiation of his or her monthly rotation]  **Hodgkinson 2017 –** [Before- and after-study clinicians received comparable prescribing and dispensing training through competency-based assessment programs], [After-study clinicians received additional training on e-prescribing provided by an e-prescribing pharmacist. This involved presentations and/or face-to-face tutorials, with completion of an online training module that required the ﬁrst time clinicians to login to the e-prescribing system], [Training to manage errors related to individual end-user practices was provided directly to the clinician involved]  **Howlett 2020 –** [Prescribing training is mandatory], [Information and training sessions were run over a number of weeks]  **Kazemi 2011 –** [Two of the authors (A.K. and A.A.) conducted group and private training sessions for the residents to teach them how to use the prescription system]  **Liao 2017 –** [Hospital wide training sessions were required for every health care provider prior to the initiation of the EHR with CPOE system. Inpatient physicians were required to complete 4 hours of training]  **Potts 2004 –** [There was a 1-month prescribing period when no data were collected to allow for CPOE implementation and training for all attendings, fellows, residents, and staff]  **Shawahna 2011 –** [After approval, doctors were given a one-day training workshop on the new electronic inpatient record and discharge medications sheet], [Newsletters contained error examples and recommendations for safe prescribing]  **Shulman 2005 –** [The new system was introduced following a program of staff training]  **Venkataraman 2016 –** [All prescribers were formally briefed and trained for using EIC by the PCCU pharmacists] | **0.91**  (91) |
| 5. Natural consequences | 5.1 Information on health consequences | - Alert the prescriber about the consequences of placing a specific medication order (e.g. patient allergy, drug-drug interaction, therapeutic duplication, contraindication) through system alerts or warnings; verbal or written information on medication errors may also be provided | **Armada 2014** – [Alerts about duplicities, potential interactions, and allergies are also available]  **Boling 2005 –** [The warning informs the clinician of the dosing error and appropriate dosing]  **Cordero 2004 –** [Numerous clinical decision support tools are integrated into the ordering pathways. These include, but are not limited to, drug allergy, drug-drug interactions, order duplication, corollary orders, weight-based dosage, maximum dosage, and drug route restriction]  **Hodgkinson 2017 –** [Active decision-support included mandatory entry of patient allergy/adverse drug reaction (ADR) status with alerts for potential medicine–medicine or medicine–medication class reactions, and moderate and severe medicine interactions]  **Kazemi 2011 –** [If the prescribed dose or frequency was not within the normal range, the DSS informed the prescriber about the appropriate dose and/or frequency by demonstrating a warning message that asked for correction]  **Mahoney 2007 –** [The CPOE system provided the prescriber with alerts such as detection of an allergy to the prescribed medication and therapeutic duplication. Drug-specific alerts were also programmed into the system to provide information such as dosage adjustment or monitoring requirements during order entry], [The first two rules implemented were to alert the physician of inappropriate therapy when metformin or colchicine was prescribed for a patient with a serum creatinine (SCr) concentration over 1.5 mg/dL]  **Shawahna 2011 –** [Doctors, nurses and pharmacists were invited to series of seminars on medication errors (including prescribing errors). Attendants were educated on error scenarios and their consequences. Throughout the study, newsletters concerning such errors and safe prescribing practice circulated the different wards of the hospital. Newsletters contained error examples and recommendations for safe prescribing] | **0.86**  (86) |
| 7. Associations | 7.1 Prompts/cues | - Provide visual on-screen alerts or pop-ups to prompt prescribers to change or adjust potentially erroneous or unsafe medication orders | **Armada 2014 –** [Alerts about duplicities, potential interactions, and allergies are also available]  **Boling 2005 –** [When a prescriber enters a dose that exceeds the ranges in the dose range checker, the prescriber is alerted with an appropriate and specific warning]  **Cordero 2004 –** [The order is evaluated through a clinical rules engine and appropriate alerts are triggered]  **Hodgkinson 2017 –** [with alerts for potential medicine–medicine or medicine–medication class reactions, and moderate and severe medicine interactions]  **Kazemi 2011 –** [If the prescribed dose or frequency was not within the normal range, the DSS informed the prescriber about the appropriate dose and/or frequency by demonstrating a warning message that asked for correction]  **Mahoney 2007 –** [The CPOE system provided the prescriber with alerts such as detection of an allergy to the prescribed medication and therapeutic duplication. Drug-specific alerts were also programmed into the system to provide information such as dosage adjustment or monitoring requirements during order entry], [The first two rules implemented were to alert the physician of inappropriate therapy when metformin or colchicine was prescribed for a patient with a serum creatinine (SCr) concentration over 1.5 mg/dL]  **Potts 2004 –** For these patients, clinical decision support provides only recent laboratory values and an alert to take renal function into account during the ordering process] | **0.86**  (86) |
| 8. Repetition and substitution | 8.1 Behavioural practice/rehearsal | - Provide classroom or individual training sessions for prescribers to work through order examples, workbooks, online modules, or system demos | **Armada 2014 –** [The staff followed a training program on the management of the computer system for 8 hours]  **Hodgkinson 2017 –** [After-study clinicians received additional training on e-prescribing provided by an e-prescribing pharmacist. This involved presentations and/or face-to-face tutorials, with completion of an online training module that required the ﬁrst time clinicians to login to the e-prescribing system]  **Howlett 2020 –** [A dual-prescribing process was put in place. This familiarised staff with the medication ordering processes on ICCA and assisted in validation of the drug file. Paper prescriptions continued to be the legal prescription until electronic prescribing went fully “live” in November 2012]  **Kazemi 2011 -** [Residents could also obtain access to a demo version of the system for further training]  **Shawahna 2011 –** [Doctors were provided handheld computers and were asked to prescribe medications onto the medication chart] | **0.80**  (80) |

| **Table AF5.2** Table of BCTs excluded from the taxonomy | | |
| --- | --- | --- |
| **Study** | **BCT clusters** | **BCT label [evidence in text]** |
| Armada 2014 | 9. Comparison of outcomes | 9.2 Pros and cons – [The staff members were invited to qualify as “poor, fair, good, or very good” a total of 17 different aspects of this technology and to point main advantages and disadvantages with respect to the previous MP method] |
| Bizovi 2002 | 8. Repetition and substitution | 8.6 Generalisation of target behaviour – [Steps were similar to those required to complete other tasks in the system and were readily learned by ED providers] |
| Hodgkinson 2017 | 1. Goals and planning  2. Feedback and monitoring | 1.6 Discrepancy between current behaviour and goal – [Training to manage errors related to individual end-user practices was provided directly to the clinician involved]  2.2 Feedback on behaviour – [Training to manage errors related to individual end-user practices was provided directly to the clinician involved] |
| Howlett 2020 | 8. Repetition and substitution | 8.6 Generalisation of target behaviour – [A dual-prescribing process was put in place. This familiarised staff with the medication ordering processes on ICCA] |
| Kazemi 2011 | 6. Comparison of behaviour | 6.1 Demonstration of the behaviour – [Residents could also obtain access to a demo version of the system for further training] |
| Mahoney 2007 | 1. Goals and planning | 1.2 Problem solving – [Another major role of the MITT was to assist with problem solving when conflicts arose between system functionality and operational and safety priorities] |
| Shawahna 2011 | 5. Natural consequences  12. Antecedents | 5.2 Salience of consequences - [Attendants were educated on error scenarios and their consequences. Throughout the study, newsletters concerning such errors and safe prescribing practice circulated the different wards of the hospital. Newsletters contained error examples and recommendations for safe prescribing]  12.5 Adding objects to the environment – [Doctors were provided handheld computers] |

| **Table AF6.3** Number of BCTs in each study and the corresponding odds ratio | | |
| --- | --- | --- |
| **Study** | **No. BCTs coded** | **Odds Ratio (OR)** |
| Ali 2010 | 3 | 0.00 [0.00, 0.01] |
| Potts 2004 | 2 | 0.00 [0.00, 0.01] |
| Hodgkinson 2017 | 8 | 0.01 [0.00, 0.01] |
| Armada 2014 | 6 | 0.01 [0.01, 0.02] |
| Venkataraman 2016 | 3 | 0.02 [0.00, 0.13] |
| Cordero 2004 | 5 | 0.03 [0.00, 0.57] |
| Liao 2017 | 2 | 0.07 [0.05, 0.09] |
| Boling 2005 | 2 | 0.24 [0.03, 1.93] |
| Bizovi 2002 | 2 | 0.29 [0.15, 0.56] |
| Shawahna 2011 | 6 | 0.29 [0.28, 0.32] |
| Kazemi 2011 | 8 | 0.46 [0.40, 0.53] |
| Mahoney 2007 | 5 | 0.48 [0.46, 0.51] |
| Shulman 2005 | 2 | 0.72 [0.53, 0.99] |
| Howlett 2018 | 3 | 0.79 [0.60, 1.04] |
| Spearman’s correlation: rs = -0.049, n=14, p=0.868 | | |


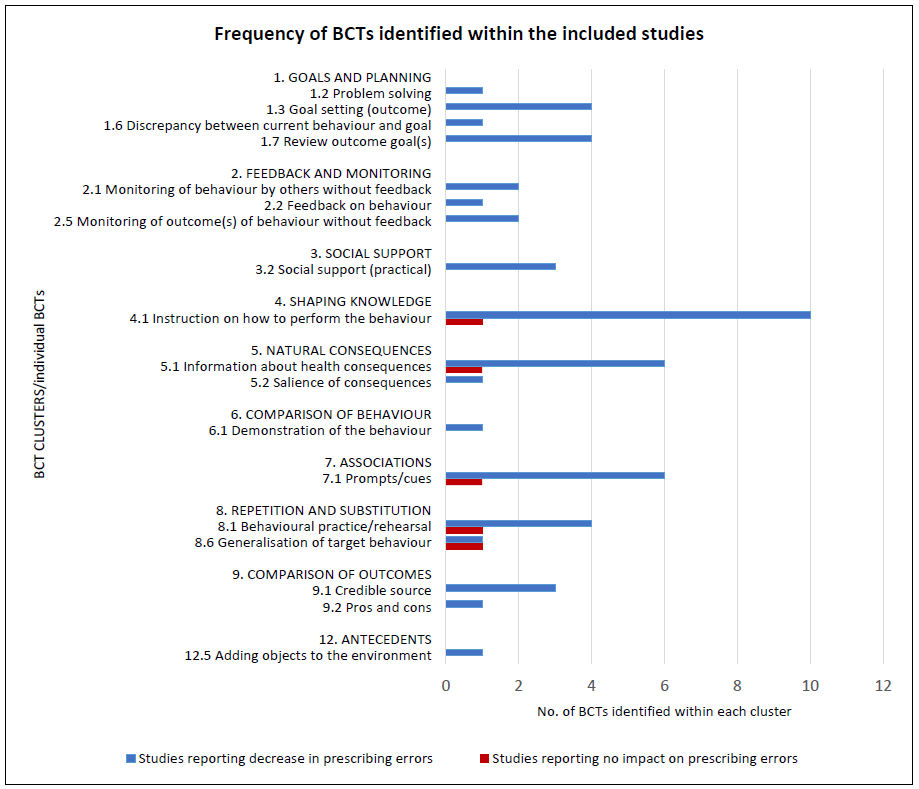


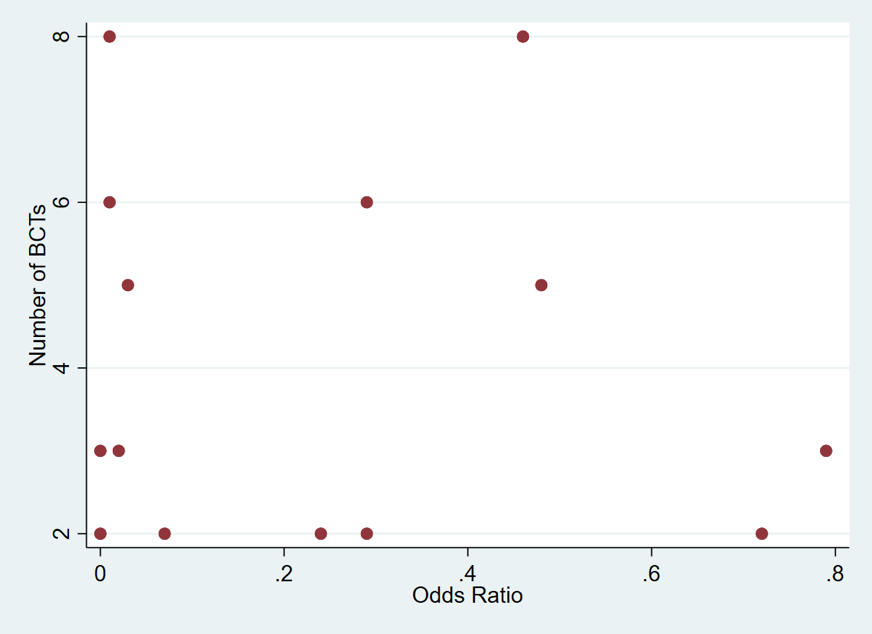


**Figure AF6.1** Number of behaviour change techniques (BCTs) by cluster coded for studies reporting a reduction in the risk of prescribing error compared to studies reporting no impact or an increase in prescribing errors

**Figure AF6.2** Twoway STATA scatterplot illustrating no association between the number of BCTs and OR of each study
